# Exploring the utility of unretouched lithic flakes as markers of cultural change

**DOI:** 10.1038/s41598-025-85399-z

**Published:** 2025-01-10

**Authors:** Manuel Will, Hannes Rathmann

**Affiliations:** 1 Department of Early Prehistory and Quaternary Ecology, University of Tübingen, 72070 Tübingen, Germany; 2 Palaeo-Research Institute, University of Johannesburg, P.O. Box 524, Auckland Park, ZA-2006 South Africa; 3 Senckenberg Centre for Human Evolution and Palaeoenvironment, University of Tübingen, 72070 Tübingen, Germany; 4 Institute for Archaeological Sciences, Department of Geosciences, University of Tübingen, 72070 Tübingen, Germany

**Keywords:** Stone tools, Palaeolithic, Stone Age, Multivariate statistics, Open science, Method development, Archaeology, Cultural evolution

## Abstract

Lithic artefacts provide the principal means to study cultural change in the deep human past. Tools and cores have been the focus of much prior research based on their perceived information content and cultural relevance. Unretouched flakes rarely attract comparable attention in archaeological studies, despite being the most abundant assemblage elements and featuring prominently in ethnographic and experimental work. Here, we examine the potential of flake morphology for tracing cultural change utilising 4,512 flakes, each characterised by 16 standard mixed-scale attributes, from a well-documented cultural sequence at the Middle Stone Age site of Sibhudu, South Africa. We quantified multivariate similarities among flakes using FLEXDIST, a highly versatile method capable of handling mixed, correlated, incomplete, and high-dimensional data. Our findings reveal a significant gradual change in flake morphology that aligns with the documented cultural succession at Sibhudu. Furthermore, our analysis provides new insights into the patterning of variability throughout the studied sequence. The demonstrated potential of flakes to track cultural change opens up additional avenues for comparative research due to their ubiquity, the availability of commonly recorded attributes, and especially in the absence of cores or tools. FLEXDIST, with its versatile applicability to complex lithic datasets, holds particular promise in this regard.

## Introduction

Stone artefacts constitute the main archaeological source for studying behavioural adaptations and material culture across ~3 million years of human evolution (e.g.^1,2^). Preserving under almost any condition, lithic artefacts remain the most continuous and abundant record available to archaeologists. Variations in the shape, size and other properties of lithic artefacts provide information on the technological organisation, settlement strategies, demographic expansions, ecological adaptations, cultural transmission, and cognitive abilities of prehistoric humans (e.g.,^3–5^). Not surprisingly, lithic artefacts have been the principal means to track cultural change across time, space and hominin species.

Not all lithic artefacts are studied equally, however. Based on their perceived information content and cultural relevance, tools and cores have been the privileged archaeological research objects, even though they are usually a minor aspect of lithic assemblages. The formative years of Palaeolithic archaeology focused almost exclusively on retouched and shaped pieces. Scholars used typologies of such tools to infer implement function and construct chrono-cultural sequences all over the world, serving as *fossils directeurs* for particular periods^6–8^. Until today, the presence of specific retouched or shaped pieces (e.g., handaxes for the Acheulean, tanged points for the Aterian or Gravette points for the Gravettian) often remains the main reason for assigning lithic remains to a specific technocomplex (see^9–11^). Since the 1980s, a methodological reorientation on the technology, functionality and reduction sequences of knapped stones has shifted the centre of attention more toward lithic assemblages in their entirety^12–15^. Still, technological or techno-functional studies frequently emphasize the roles of cores and tools, utilising their own technological typologies^16,17^. Qualitative studies tend to single out a fraction of ‘desired’ products within larger lithic assemblages such as bifaces and blades to identify specific reduction strategies via rare diagnostic pieces. Even quantitative studies, such as phylogenetic approaches based on attributes or shape analyses via geometric morphometrics, often focus on retouched/shape elements or core categories^18–20^.

Unretouched flakes, either in their entirety or as an analytical unit, rarely take the centre spot of archaeological studies (but see^5,21–23^), despite their documented importance in ethnographic work^17,24^ and being a focus in experimental research^25–27^. Nonetheless, flakes constitute the most prevalent pieces in lithic assemblages throughout the global Palaeolithic and Stone Age records, greatly surpassing cores and tools in abundance. The ‘flake’ category may encompass a large variability of pieces struck from a core, generally without specific shapes or sizes, and includes a multitude of different items from the decortication, preparation, maintenance and production phases. Flake assemblages may or may not contain ‘diagnostic pieces’ indicative of specific technologies such as bifacial shaping from handaxe production, or Levallois flakes and by-products. Drawing on assemblages from the Middle Palaeolithic, however, Archer et al.^23^ find that in most assemblages only <25% of all flakes can be attributed to a specific technological system. The qualitative identification of such technological types remains a rather coarse-grained instrument, dependent on expert knowledge and with low levels of inter-observer replicability (see^23,28–30^). These classifications are used in conjunction with cores to identify technological systems such as shaping vs. debitage, Levallois vs. volumetric cores, or to differentiate between periods such as the Lower and Middle Palaeolithic^12–14^. More recently, quantitative approaches employing attribute analyses on flakes have been used to identify reduction sequences and technological types based on statistical analyses^21,23,31,32^. These studies remain few and usually do not focus on tracking cultural change through time within sites or across technocomplexes. Finally, although numerous continuous-, ordinal-, and nominal-scale attributes are routinely recorded on flakes as part of standard lithic data collection protocols, many archaeological studies or site reports restrict their flake analyses to a tabulation of flake numbers or summary statistics of selected attributes.

To neglect unretouched flakes in assessments of past cultural change might unnecessarily hinder Palaeolithic archaeology from harnessing the full information content existent in these assemblage elements. The common presence, large number and high variability in multiple dimensions also render flakes and their attributes particularly amenable to statistical analyses on multiple scales and ideal for comparative purposes in a unified framework (see^5,22,23,29,30^). Do unretouched flakes carry less relevant information on past human behaviours? Can we use such elements to track cultural change within sites or technocomplexes in the absence of cores, tools and ‘diagnostic’ pieces?

Here, we examine the potential of unretouched flakes for tracing cultural change in a test study utilising a large sample of flakes from the Middle Stone Age (MSA) site of Sibhudu, located in South Africa. Sibhudu presents an ideal test case, benefitting from many years of research that has established a well-dated, extensive stratigraphic sequence with abundant data from lithic analyses reflecting a known succession of cultural changes (see below). Flakes number into the many thousands and are recorded with multiple standard continuous and discontinuous variables. We restricted our analysis here to flakes based on the arguments raised above as well as disagreements of definitions for blades and bladelets that may diminish the replicability of this study design. If successful on flakes alone, the addition of elongated, parallel shapes in subsequent studies is likely to increase the resolution and robustness of the presented proof of method due to an increase in morphological variance. In the following, we quantitatively assess the morphological similarities among flakes within the site’s sequence using the newly developed multivariate FLEXDIST approach^33^. FLEXDIST offers high versatility for estimating relatedness among objects in complex datasets, due to its ability to accommodate multiple variable scales (i.e., continuous, ordinal, nominal, or any combination thereof), account for variable correlations, handle missing values, and manage high-dimensional datasets. FLEXDIST was initially devised for analysing skeletal morphological variation but is, in principle, applicable to any other mixed, correlated, fragmented, and high-dimensional data, regardless of being biological or cultural in nature. This work aims to achieve two interconnected objectives: firstly, to test the ability of flakes to track cultural change and, secondly, to showcase the suitability of FLEXDIST for analysing lithic artefacts.

## Results

Sibhudu constitutes one of the key sequences of the MSA in South Africa with stratified deposits of occupations rich in archaeological material left by modern humans in a long sequence dating to >100-38 ka^34–36^. We previously studied the large lithic assemblages of the so-called ‘Sibhudan’ sequence in this site^37–39^. This part of the Sibhudu deposits reaches 1.2 m in thickness. It features a total of 23 archaeological layers, with both the top and bottom strata dated by Optically Stimulated Luminescence to ~58 ka^40^. The large number of lithic artefacts, highly resolved chronology and similar residential character of the occupations within this sequence in comparable ecological conditions provided an ideal setting for identifying short-term diachronic cultural change at a site and within a technocomplex and minimized other factors that may also influence lithic technology (e.g., environmental changes, site function or raw material availability; see^37–39^ for further discussion of these aspects at Sibhudu). The original quantitative and qualitative study of all assemblage elements by a single observer (*n*=10,882) showed directional change over the sequence in various lithic domains, including directed variation in core reduction, blank production and tool manufacture. This work identified a total of four phases (1-4) that exhibit some common techno-typological features and gradually build upon another (see Supplementary Note S1, Supplementary Table S1 and Supplementary Table S2). These phases and the unidirectional trends identified in the sequence constitute our baseline expectation based on prior information for cultural change in this study.

To test whether unretouched flakes can in principle track this trend and reconstruct patterns observed in previous work based on a much larger set of lithic artefacts and contextual information, we apply FLEXDIST to a sub-sample of flakes from the Sibhudu sequence (*n*=4,512). This selection of flakes can be assigned to four successive cultural phases: phase 1 (top, *n*=761); phase 2 (*n*=1,689); phase 3 (*n*=594); and phase 4 (bottom; *n*=1,468). For each flake, we selected 16 variables (Table 1). These include, for instance, continuous-scale length and width measurements, ordinal-scale relative expression levels of bulbs, or nominal-scale presence or absence states of proximal lips. In our main analysis, we allowed for some missing data per flake (with a maximum threshold set arbitrarily at 20%), which considerably increased the available sample size at Sibhudu compared to analysing complete flakes only. Five continuous measurements characterising the overall geometric properties of the flakes (length, width, thickness, platform width, and platform thickness) were converted into scale-free shape variables to facilitate comparisons among flakes of different sizes but similar shapes.

**Table 1:** List of variables employed to characterise variation in unretouched lithic flakes at Sibhudu, along with their abbreviations, measurement scales, levels, and summary statistics.

Variable	Abbreviation	Scale (Levels)	Summary Statistics
Raw material	RMA	Nominal (1 = Dolerite; 2 = Hornfels; 3 = Sandstone; 4 = Quartzite; 5 = Quartz)	1 = 3189; 2 = 263; 3 = 638; 4 = 250; 5 = 172; NA´s = 0
Percent cortex	CPE	Continuous	Min. = 0; 1st Qu. = 0; Median = 0; Mean = 12.35; 3rd Qu. = 20; Max. = 100; NA´s = 2
Length	LEN	Continuous	Min. = 8; 1st Qu. = 27; Median = 32; Mean = 34.06; 3rd Qu. = 39; Max. = 129; NA´s = 1358
Width	WID	Continuous	Min. = 6; 1st Qu. = 24; Median = 29; Mean = 30.59; 3rd Qu. = 35; Max. = 114; NA´s = 837
Thickness	THI	Continuous	Min. = 2; 1st Qu. = 6; Median = 8; Mean = 8.69; 3rd Qu. = 10; Max. = 38; NA´s = 42
Exterior Platform Ange	EPA	Continuous	Min. = 35; 1st Qu. = 79; Median = 84; Mean = 82.22; 3rd Qu. = 88; Max. = 105; NA´s = 305
Shape	SHA	Nominal (1 = Convergent; 2 = Divergent; 3 = Round/oval; 4 = Parallel/sub-parallel; 5 = Rectangular)	1 = 725; 2 = 675; 3 = 331; 4 = 742; 5 = 922; NA´s = 1117
Bulb	BUL	Ordinal (1 = Absent; 2 = Poor-developed; 3 = Developed; 4 = Shattered; 5 = Well-developed)	1 = 692; 2 = 1107; 3 = 1200; 4 = 996; 5 = 476; NA´s = 41
Eraillure scar	ESC	Nominal (0 = Absent; 1 = Present)	0 = 4353; 1 = 159; NA´s = 0
Lip	LIP	Nominal (0 = Absent; 1 = Present)	0 = 4149; 1 = 363; NA´s = 0
Hertzian cone	HCO	Nominal (0 = Absent; 1 = Present)	0 = 4386; 1 = 126; NA´s = 0
Platform type	PTY	Nominal (1 = Cortical; 2 = Plain; 3 = Facetted; 4 = Dihedral; 5 = Crushed/shattered; 6 = Linear/punctiform)	1 = 302; 2 = 2958; 3 = 505; 4 = 364; 5 = 251; 6 = 85; NA´s = 47
Platform width	PWI	Continuous	Min. = 1; 1st Qu. = 13; Median = 17; Mean = 18.51; 3rd Qu. = 23; Max. = 76; NA´s = 490
Platform thickness	PTH	Continuous	Min. = 1; 1st Qu. = 4; Median = 6; Mean = 6.41; 3rd Qu. = 8; Max. = 36; NA´s = 142
Number of negatives	NNU	Ordinal (0 = 0, 1 = 1, 2 = 2, 3 = 3, 4 = 4, 5 = 5, 6 = 6, 7 = 7, 8 = 8, 9 = 9)	0 = 75; 1 = 487; 2 = 1256; 3 = 1500; 4 = 804; 5 = 289; 6 = 70; 7 = 18; 8 = 3; 9 = 1; NA´s = 9
Orientation of negatives	NOR	Nominal (1 = Parallel; 2 = Opposed; 3 = Convergent; 4 = Orthogonal; 5 = Centripetal)	1 = 1281; 2 = 504; 3 = 153; 4 = 1024; 5 = 820; NA´s = 730

**Fig. 1:**
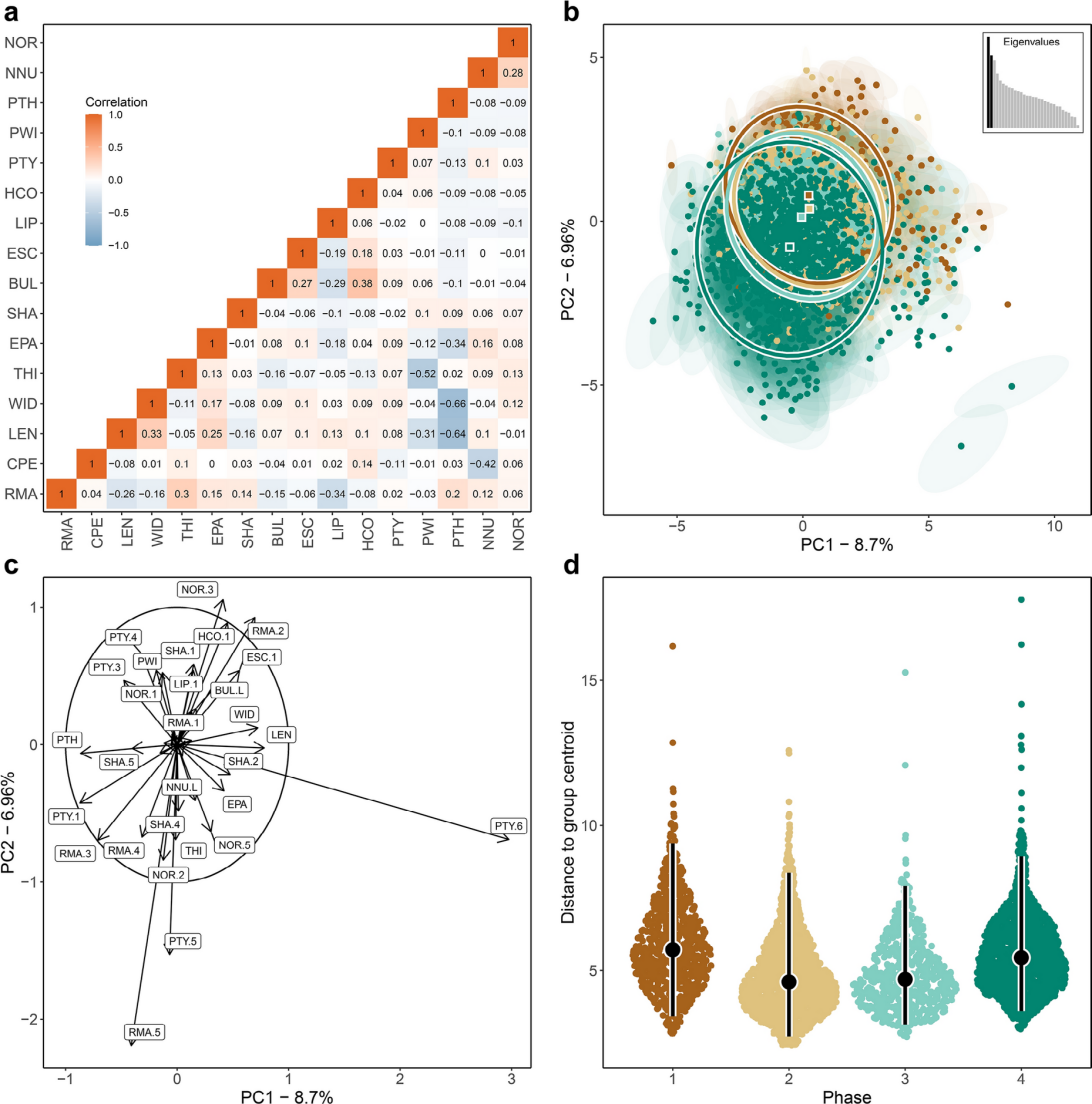
Morphological resemblance among 4,512 unretouched lithic flakes from four successive cultural phases at Sibhudu, with each flake characterised by 16 variables and allowing for up to 20% missing data. (**a**): Pairwise correlations among variables, with colour gradients indicating the direction and strength of each correlation. Full variable names and abbreviations are provided in Table 1. Five continuous measurements characterising the overall flake geometry (LEN = length, WID = width, THI = thickness, PWI = platform width, and PTH = platform thickness) were converted into scale-free shape variables to enable comparisons among flakes of different sizes but similar shapes. (**b**): PCA plot generated by FLEXDIST_plot_, illustrating the multivariate distribution of flakes (depicted as dots) in two-dimensional PC space. Each flake is surrounded by a 95% confidence ellipse, displaying uncertainty resulting from missing data; larger ellipses indicate more missing values, while smaller ellipses indicate fewer missing values. Color-coding denotes the cultural phase attribution of each flake (see panel d). For each phase, a centroid estimate marks the central location (depicted as a square), accompanied by a 95% confidence ellipse illustrating the dispersion around the centroid. The inset box displays a bar plot of eigenvalues, where bars denote the variance explained by each PC, with the variance retained by the first two PCs indicated in black. (**c**): PCA correlation circle plot visualizing how much the original variables are correlated with the first two PCs. Each variable is represented by an arrow emanating from the centre. Arrows pointing close to the principal axes suggest that the variable is well-represented by that particular PC, while the length of the arrows indicates how much each variable contributes to the PCs. (**d**): Sina plots showing the dispersion of flakes within the four phases, estimated as the distance of each flake to the respective phase centroid. Error bars are superimposed on the distributions to display medians (depicted as dots) and 95% interpercentile ranges (depicted as bars). This figure was generated in R using the code provided in Supplementary Information Code S1.

As an initial step, we examined the correlation structure of the 16 mixed continuous, ordinal, and nominal variables by computing a heterogeneous correlation matrix (Figure 1a). Overall, we found a high degree of independence among most variables, with a few notable exceptions where stronger associations were observed. For example, we found a positive correlation between the expression of the bulb and the presence of an Hertzian cone. Similarly, platform thickness showed a negative correlation with both the length and width of the flakes when corrected for size, though this changes to a positive correlation without size correction (Supplementary Figure S1a). These associations are well-documented in existing literature on experimental flaking (summary in^27^) and need to be taken into account analytically when estimating multivariate resemblance among flakes to avoid over-representing variation from variables that co-occur. Contrary to our expectations, raw material was not strongly correlated with most attribute variables in the dataset, potentially due to the dominance of dolerite (71%) in the total flake assemblage.

**Fig. 2:**
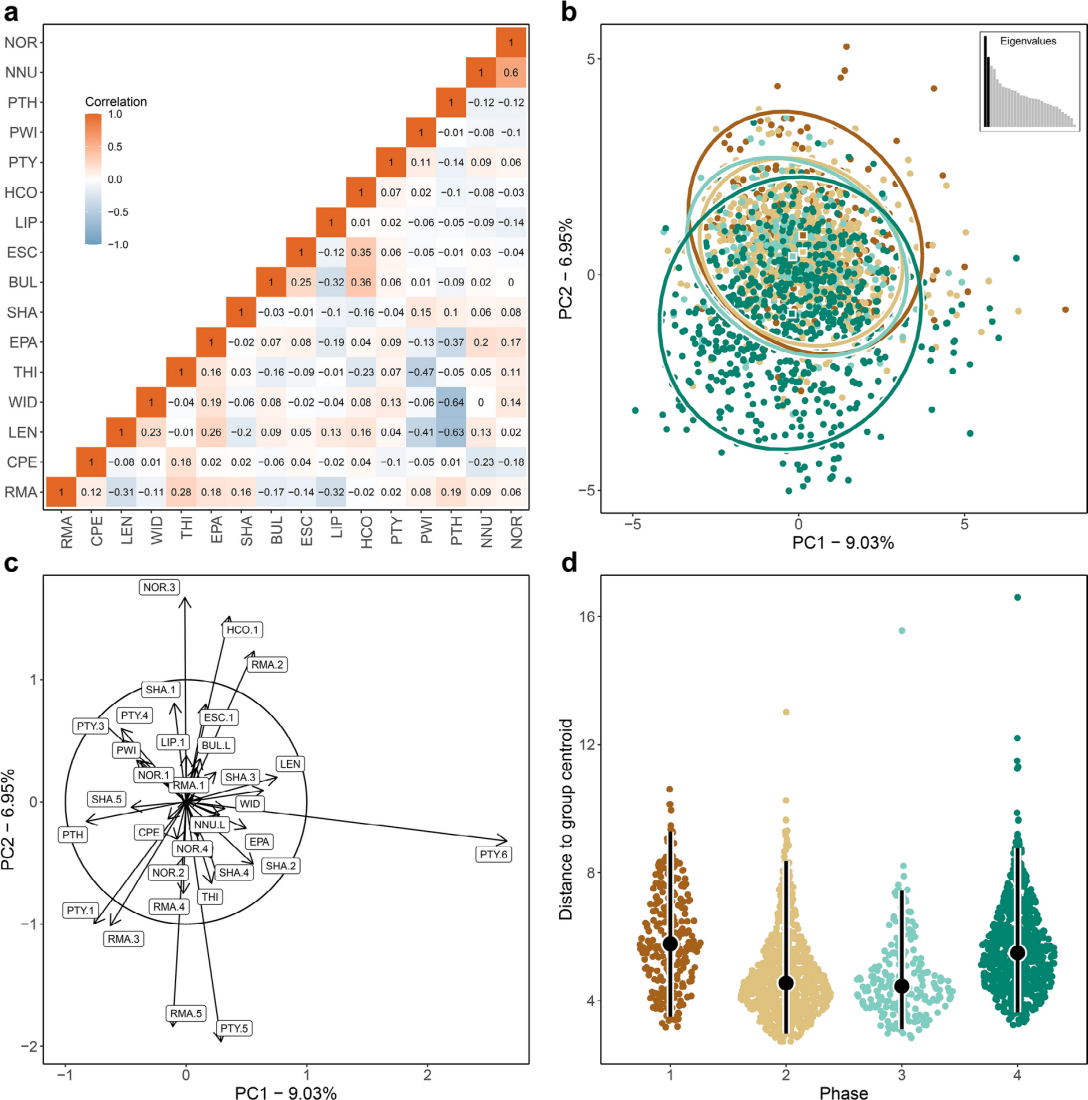
Morphological resemblance among 1,877 unretouched lithic flakes from four successive cultural phases at Sibhudu, with each flake characterised by 16 variables and no missing data. (**a**): Pairwise correlations among variables, with colour gradients indicating the direction and strength of each correlation. Full variable names and abbreviations are provided in Table 1. Five continuous measurements characterising the overall flake geometry (LEN = length, WID = width, THI = thickness, PWI = platform width, and PTH = platform thickness) were converted into scale-free shape variables to enable comparisons among flakes of different sizes but similar shapes. (**b**): PCA plot generated by FLEXDIST_plot_, illustrating the multivariate distribution of flakes (depicted as dots) in two-dimensional PC space. Each flake is surrounded by a 95% confidence ellipse, displaying uncertainty resulting from missing data; larger ellipses indicate more missing values, while smaller ellipses indicate fewer missing values. Color-coding denotes the cultural phase attribution of each flake (see panel d). For each phase, a centroid estimate marks the central location (depicted as a square), accompanied by a 95% confidence ellipse illustrating the dispersion around the centroid. The inset box displays a bar plot of eigenvalues, where bars denote the variance explained by each PC, with the variance retained by the first two PCs indicated in black. (**c**): PCA correlation circle plot visualizing how much the original variables are correlated with the first two PCs. Each variable is represented by an arrow emanating from the centre. Arrows pointing close to the principal axes suggest that the variable is well-represented by that particular PC, while the length of the arrows indicates how much each variable contributes to the PCs. (**d**): Sina plots showing the dispersion of flakes within the four phases, estimated as the distance of each flake to the respective phase centroid. Error bars are superimposed on the distributions to display medians (depicted as dots) and 95% interpercentile ranges (depicted as bars). This figure was generated in R using the code provided in Supplementary Information Code S1.

In the second step, we quantified the multivariate morphological resemblance among flakes using FLEXDIST. Essentially, FLEXDIST extends a conventional principal component analysis (PCA) to accommodate mixed data types, iterating over multiple randomly imputed datasets to account for uncertainty arising from incomplete cases. It decomposes a large set of possibly correlated variables into a smaller set of new uncorrelated variables, called principal components (PCs). The goal is to reduce the dimensionality of the data while retaining the most important aspects of their variation. The first two PCs that explain most of the variation across objects are visualized in a two-dimensional scatterplot (FLEXDIST_plot_), where each object is represented by the centroid estimate of its various imputed duplicates. A 95% confidence ellipse surrounding the centroid illustrates the uncertainty resulting from missing data. Like PCA, FLEXDIST is a purely exploratory tool, useful for identifying patterns in the data, but it does not explicitly test any predefined hypotheses. Figure 1b presents FLEXDIST_plot_, illustrating the resemblance among lithic flakes belonging to the four cultural phases at Sibhudu. The first two PCs, which capture the most important aspects of variation in the data, together explain only 15.7% of the total variation, leaving the remaining 84.3% unexplored among the PCs not shown. Overall, the plot reveals an expected high degree of resemblance across all analysed flakes, with a large amount of overlap observed between the four phases. However, along PC2, a discernible temporal pattern emerges, where the central location of the cloud of flakes for each phase, known as centroids, sequentially differs from one phase to another in accordance with their succession in the sequence. Additionally, the spread of the cloud of flakes in each phase, known as dispersion, indicates different levels of intra-phase variation, with phase 1 and 4 appearing more heterogeneous than phase 2 and 3.

In the third step, we used the loadings provided by FLEXDIST to generate a PCA correlation circle plot (Figure 1c), providing insights into the correlation between the original variables and the first two PCs. This plot helps in interpreting how the original variables relate to the structure captured by these two PCs, making it easier to identify the key drivers of variation. Each variable from the dataset is represented by an arrow emanating from the centre of the plot. Arrows pointing close to the principal axes suggest that the variable is well-represented by that particular PC, while the length of the arrows indicates how much each variable contributes to the PCs. The angle between arrows shows correlations among variables; arrows pointing in the same direction indicate positive correlation, whereas arrows pointing in opposite directions suggest negative correlation. By examining this plot, we found that variation along PC1 is primarily attributed to the platform type ‘linear/punctiform’. Conversely, variation along PC2 is primarily driven by a combination of the raw material quartz and the platform type ‘crushed/shattered’ on the one hand, and by a combination of the convergent orientation of dorsal negatives and the raw material hornfels on the other.

In the fourth step, we further examined the differences in intra-phase dispersion which we visually identified in Figure 1b. To achieve this, we once again utilised the information provided by FLEXDIST to estimate the distance of each flake centroid to the corresponding group centroid of each phase. We then visualized the resulting distributions of these distance values using sina plots (Figure 1d). In these plots, lower overall values indicate greater homogeneity, while higher overall values reflect increased heterogeneity. The plotted distributions reveal a reduction in intra-phase dispersion from phase 1 to phase 2, a consistent level of dispersion between phases 2 and 3, and an increase in dispersion from phase 3 to phase 4.

In the fifth and final step, we formally tested hypotheses regarding differences in phase centroids and dispersion, employing statistical inference techniques that utilise the entire high-dimensional space. This approach contrasts sharply with the visual inspection of FLEXDIST_plot_ in Figure 1b, which depicts variation only along the first two PCs, and may therefore overlook more complex differences that exist in higher dimensions beyond those represented by the first two PCs. To statistically corroborate the visually inferred shift in centroids, we conducted a permutational multivariate analyses of variance (PERMANOVA)^41^, using the FLEXDIST flake centroid estimates (Table 2). PERMANOVA explicitly tests the null hypothesis of no differences in the centroids among phases. Pairwise PERMANOVA testing confirmed our visual interpretations (Figure 1b) and revealed highly significant differences in centroids across all four phases. Next, to statistically validate the visually inferred differences in dispersion, we employed a permutational multivariate analysis of dispersion (PERMDISP)^42^, again based on the FLEXDIST flake centroid estimates (Table 2). PERMDISP explicitly tests the null hypothesis of no differences in dispersion among groups. Pairwise PERMDISP testing confirmed our visual inspections (Figure 1d) and revealed highly significant differences in dispersions among all phases, except for the pairwise comparison between phases 2 and 3.

**Table 2:** Pairwise PERMANOVA and PERMDISP tests conducted on 4,512 unretouched lithic flakes from four cultural phases at Sibhudu, with each flake characterised by 16 variables and allowing for up to 20% missing data.

	PERMANOVA					PERMDISP				
Pairs	*n*	*df*	*F*	*R*^*2*^	*P*	*n*	*df*	*F*	*R*^*2*^	*P*
Phase 1 vs. 2	1,522	1; 1,520	17.2198	0.0112	0.0009*	1,522	1; 1,520	162.6583	0.0967	0.0009*
Phase 1 vs. 3	1,188	1; 1,186	17.0855	0.0142	0.0009*	1,188	1; 1,186	133.0673	0.1009	0.0009*
Phase 1 vs. 4	1,522	1; 1,520	33.4867	0.0216	0.0009*	1,522	1; 1,520	19.2646	0.0125	0.0009*
Phase 2 vs. 3	1,188	1; 1,186	4.1365	0.0035	0.0009*	1,188	1; 1,186	0.1140	< 0.0000	0.7303
Phase 2 vs. 4	2,936	1; 2,934	48.8571	0.0164	0.0009*	2,936	1; 2,934	193.7419	0.0619	0.0009*
Phase 3 vs. 4	1,188	1; 1,186	15.4119	0.0128	0.0009*	1,188	1; 1,186	74.6916	0.0592	0.0009*

*n*=sample size; equal sample sizes were ensured by randomly down-sampling the larger sample to match the size of the smaller one; *df*=model and residual degrees of freedom; *F*=*F* test statistic; *R*^*2*^=coefficient of determination; *P*=*P*-value obtained using 1,000 permutations; *=statistical significance after Bonferroni correction for multiple testing.

To evaluate the extent to which our results were affected by the inclusion of flakes with missing data, we re-ran the entire analyses, this time focusing exclusively on complete flakes. This reduced the number of analysable flakes from *n*=4,512 to *n*=1,877, with the four successive cultural phases of the Sibhudan sequence represented as follows: phase 1 (*n*=245); phase 2 (*n*=717); phase 3 (*n*=214); and phase 4 (*n*=701). Despite the smaller sample size, the resulting patterns closely aligned with those observed previously (Figure 2), albeit with marginally higher PERMANOVA and PERMDISP *R*^*2*^-values overall (Table 3). This indicates a slightly higher proportion of flake variability explained by the differences between the phases. The only notable difference was a non-significant PERMDISP *P*-value for the pairwise comparison between phases 1 and 4 in the subsample of complete flakes, whereas this comparison had been significant in the larger dataset that included incomplete flakes. However, given the similar *R*^*2*^ values across both analyses, this discrepancy is likely due to the more than threefold reduction in sample size when analysing only complete flakes.

**Table 3:** Pairwise PERMANOVA and PERMDISP tests conducted on 1877 unretouched lithic flakes from four cultural phases at Sibhudu, with each flake characterised by 16 variables with no missing data.

	PERMANOVA					PERMDISP				
Pairs	*n*	*df*	*F*	*R*^*2*^	*P*	*n*	*df*	*F*	*R*^*2*^	*P*
Phase 1 vs. 2	490	1; 488	7.5991	0.0153	0.0009*	490	1; 488	64.8418	0.1173	0.0009*
Phase 1 vs. 3	428	1; 426	8.8100	0.0203	0.0009*	428	1; 426	82.2256	0.1618	0.0009*
Phase 1 vs. 4	490	1; 488	11.6422	0.0233	0.0009*	490	1; 488	5.6979	0.0115	0.0090
Phase 2 vs. 3	428	1; 426	2.5543	0.0060	0.0009*	428	1; 426	0.1814	0.0004	0.6703
Phase 2 vs. 4	1,402	1; 1,400	24.3586	0.0171	0.0009*	1,402	1; 1,400	103.1832	0.0686	0.0009*
Phase 3 vs. 4	428	1; 426	6.6206	0.0153	0.0009*	428	1; 426	46.4950	0.0984	0.0009*

*n*=sample size; equal sample sizes were ensured by randomly down-sampling the larger sample to match the size of the smaller one; *df*=model and residual degrees of freedom; *F*=*F* test statistic; *R*^*2*^=coefficient of determination; *P*=*P*-value obtained using 1,000 permutations; *=statistical significance after Bonferroni correction for multiple testing.

To enable comparisons between flakes of similar shapes but different sizes, we converted five continuous measurements describing the flakes’ overall geometry into scale-free shape variables for all prior analyses. To assess the impact of this adjustment, we re-analysed the dataset without applying this correction. As expected, the uncorrected measurements showed, on average, stronger positive correlations with one another (Supplementary Figure S1a). Additionally, the two-dimensional FLEXDIST_plot_ revealed a greater degree of overlap between the four phases (Supplementary Figure S1b), attributed to the relatively stronger contribution of the uncorrected measurements along PC1 (Supplementary Figure S1c). Despite these differences, both the PERMANOVA and PERMDISP tests, which assessed the full high-dimensional space beyond the first two PCs depicted in FLEXDIST_plot_, yielded results nearly identical to those obtained with the correction applied (Supplementary Table S3).

Lastly, our results may be influenced by the inclusion of raw material as an attribute, as it may drive much of the observed variation across cultural phases, as also partially suggested in Figure 1c and Figure 2c. Consequently, this attribute could overshadow the more subtle contributions of other explanatory variables to the overall variation in lithic flakes. In fact, when we re-ran our analysis using raw material as the grouping variable instead of phase, we found highly significant PERMANOVA results, along with some significant PERMDISP results (Supplementary Figure S2, Supplementary Table S4). To address this issue, we conducted two additional sets of more cautious analyses; one excluding raw material as an explanatory variable (Supplementary Figure S3, Supplementary Table S5) and another focusing solely on a single raw material which was sufficiently available across all four phases, namely, dolerite (Supplementary Figure S4, Supplementary Table S6). The latter analysis reduced the number of analysable flakes from *n*=4,512 to *n*=3,189, with the four successive cultural phases of the Sibhudan sequence represented as follows: phase 1 (*n*=530); phase 2 (*n*=1,552); phase 3 (*n*=503); and phase 4 (*n*=604). In both analyses, the resulting two-dimensional FLEXDIST_plot_ showed substantial overlap, with no visible distinctions between phases on the plot. However, the PERMANOVA and PERMDISP tests, which explored the full high-dimensional space, revealed patterns generally consistent with those observed when raw material was included as an explanatory variable, though with lower *R*^*2*^-values on average. This suggests a reduced proportion of flake variability explained by the differences between phases when raw material is excluded. Yet the results remain meaningful and robust, reproducing all statistically significant differences observed in analyses that included raw material.

## Discussion

Our study used the recently developed software tool FLEXDIST^33^ to test whether the morphology of unretouched flakes, captured in multiple continuous and discontinuos attributes, can effectively track previously identified cultural changes within the MSA sequence of Sibhudu^37–39^. Our analysis revealed a gradual change in flake morphology, with notable distinctions observed from one phase to the next, aligning with the temporal succession of phases within the sequence. This pattern is evident in the visually identified sequential shift of phase centroid estimates in two-dimensional space (Figure 1b and Figure 2b). It is further statistically corroborated by pairwise PERMANOVA testing of the entire high-dimensional space, which revealed significant differences in centroids among all four phases (Table 2 and Table 3). Some of these differences are influenced by variations in raw material usage, but not all. The results underscore the high information content and sensitivity of flakes to retain information on past cultural change on relatively small diachronic scales and within one site. Our findings have wider ramifications for general lithic analyses but also the application of multivariate statistical tools to the quantitative study of stone artefacts.

Flakes constitute the most frequent and temporally continuous element of lithic assemblages throughout the global Palaeolithic, making them an ideal target for statistical and comparative research. Yet, many previous stone artefact studies, both technological and typological, have privileged specific tools, cores or other ‘diagnostic’ pieces on the assumptions of their high information content for inferring cultural processes (see also on this issue^17^). Here we show that in principle, flake assemblages alone can be used to trace cultural change across short time scales and within sites, opening up an additional avenue for research. These results are particularly relevant for localities and sequences that do not or only rarely feature cores or tools. Even when a good number of such pieces are present, the sheer abundance and inherent variability in flake assemblages can provide important additional information.

An advantage of this and other quantitative approaches to lithic artefact variation lies in their reliance on minimal interpretative assumptions, rendering them more objective and replicable. The process of first identifying and then assigning special value to particular assemblage elements, as commonly performed in qualitative and technological approaches, inherently introduces subjective judgment and necessitates expert knowledge. Both aspects can bias the eventual outcomes and may hinder large-scale comparative research^23,28–30^. Applying FLEXDIST to flake assemblages enables researchers to study all available flakes and include all available continuous-, ordinal-, and nominal-scale attributes commonly recorded, without the need to focus solely on a subset of attributes or pieces. The incorporation of missing values greatly increases sample sizes by the inclusion of broken pieces, such as proximal fragments, which are frequent elements in Palaeolithic assemblages. Comparative research and replicability are key aspects in science and are becoming increasingly relevant for lithic analyses in the 21st century (e.g.,^29,43^). In conjunction with recent studies on the replicability of individual attributes on flakes^30,44,45^ and considering that flakes are the most frequent and common lithic products, FLEXDIST allows for the systematic comparison of flake assemblages across and within sites, regions and even across continents if comparable attribute definitions and recording procedures are followed. These attribute data already exist for many Palaeolithic sites. FLEXDIST can add new value to these large amounts of existing attribute information, opening the door to further insights into processes such as cultural transmission or demographic expansion across regions or within technocomplexes.

What do these results add to previous work on the MSA sequence of Sibhudu? On top of supporting the prior techno-typological assessment of the entire assemblages and contextual elements such as natural stratigraphic order and lithic densities (see^37–39^, and Supplementary Note S1), FLEXDIST provided new insights into the patterning of flake variation throughout the sequence. Specifically, we found different levels of variability within the four phases, with a reduction of intra-phase variation from phase 1 to 2, a similar level of variation between phases 2 and 3, and an increase in variation from phase 3 to 4. This pattern is evident in the visually identified distribution of flakes in two-dimensional space (Figure 1b and Figure 2b), the plotted distances of each flake to the respective phase centroid (Figure 1d and Figure 2d), and is further statistically corroborated by pairwise PERMDISP testing of the entire high-dimensional space (Table 2 and Table 3). The significantly higher variability of flakes in phases 1 and 4 compared to phases 2 and 3 can be contextualised with previous technological data. The dominance of one core reduction method for the production of specific pieces (i.e., discoid in phase 2 and Levallois in phase 3) associated with intense use of dolerite (73-94%) was previously observed, fitting with comparatively low variability. Phase 1 is characterised by a larger variety of raw materials, core reduction methods and resulting flake shapes. The more variable flake shapes of phase 4 may result from the common use of quartz and sandstone combined with mostly opportunistic platform but also bipolar methods. The study of all flakes and 16 attributes within a single analysis also identified the main drivers of change across the sequence (Figure 1c and Figure 2c): key flake attributes include the types of platforms, the orientation of dorsal negatives and raw materials. The latter two echo the previous quantitative and qualitative technological analyses that found a stronger production of convergent flakes and associated patterns of core reduction in phase 1 and 2 and a major shift in raw materials from abundant hornfels in phase 1 to a higher preference for quartz and sandstone, and an absence of hornfels in phase 4. This supports general observations that raw materials constitute one of the main factors influencing lithic technology^46–49^ and resonates with our findings on using raw material as a grouping variable which found significant statistical differences (Supplementary Figure S2, Supplementary Table S4). While also being a cultural choice, the specific knapping and functional properties of different raw materials (e.g.,^50,51^) might mask underlying cultural change. That being said, when excluding the raw material variable from the analyses (Supplementary Figure S3, Supplementary Table S5), or when focusing solely on dolerite (Supplementary Figure S4, Supplementary Table S6), our study was able to reproduce the same sequence and main trends as described before, underscoring the efficacy of flakes in tracing cultural change and the robustness of the FLEXDIST analysis across a range of rock types.

Our findings carry important methodological implications for quantitative analyses of lithic artefact variation in general and the application of FLEXDIST in particular. Researchers commonly rely on continuous measurements when quantitatively analysing cultural change through lithic artefacts using a PCA design or similar multivariate techniques. Typically, these measurements take the form of linear dimensions obtained with hand-held sliding callipers or more specialized instruments thereof, such as the Crossbeam Co-ordinate Calliper^52^. In the last decade, advancements in non-contact scanning techniques have enabled the generation of highly detailed visual models of lithic artefacts. 2D and 3D geometric morphometric analyses based on Cartesian landmark and semi-landmark coordinates are increasingly favoured for their ability to more accurately preserve the geometry and outline of studied objects compared to conventional calliper-based measurements^23,53^. These approaches are valuable and have allowed for high accuracy and replicability in assessing the shapes of stone artefacts in various Palaeolithic contexts^23,54–56^. All these approaches are restricted, however, to evaluating the overall continuous form, shape and size of the studied objects, but they neglect important discontinuous, qualitative features of lithic variation, such as platform types, raw materials or the presence/absence of lips. Incorporating these additional aspects of lithic variation requires more holistic approaches and statistics that account for variables measured on different scales. One widely used multivariate measure of resemblance suitable for a mixture of variable scales is the Gower similarity coefficient^56^. This coefficient can process multiple variable scales (i.e., continuous, ordinal, nominal, or any combination thereof), handles missing values, and allows for high-dimensional data. The primary limitation of the Gower coefficient, however, is its assumption of complete independence among variables. This issue poses challenges when analysing the highly correlated nature of many lithic attributes that are well-known from controlled experimental work (e.g.,^25–27^). This may explain why, to the best of our knowledge, no lithic analyses have utilised the Gower coefficient for quantifying cultural change. FLEXDIST was specifically developed as an effective alternative to the Gower coefficient, capable of considering correlations among variables. Using simulations, Rathmann et al.^33^ demonstrated that FLEXDIST generally outperforms the Gower coefficient in various artificially generated mixed data scenarios, regardless of the percentage of missing values or the dimensionality of the data.

Given the versatility of FLEXDIST and our findings from Sibhudu that show the principal applicability of the method to lithic artefacts, we suggest that FLEXDIST may prove valuable for a range of cultural datasets in future archaeological research endeavours, including ceramic or metal objects. The fully automated, open-source R code for FLEXDIST is publicly available (https://doi.org/10.5281/zenodo.10591665) allowing for easy access, method replicability and potential improvements. These are essential features for strengthening open science and collaborative approaches among researchers in archaeology and lithic analysis^29,45,57–61^. To evaluate the wider applicability, impact and limits of FLEXDIST, additional studies will need to test the method on flake assemblages from other site sequences with more complex patterns of cultural change, different periods, site function, technological backgrounds and raw materials. Future work could also check different numbers and combinations of attributes, informed by experimental studies and used for a specific focus such as patterns of cultural transmission (e.g.^5,59,62,63^). Unretouched blades and bladelets could also be included in future studies, which may result in even clearer patterns of variation than identified here due to their marked shape differences. Limits to such studies lie in systematically recording multi-scale attribute data on flakes and the replicability of such observations when multiple analysts are involved^30,44,45^. 21st century lithic analysis attests to a shift towards recording more replicable quantitative data, pursuing collaborative research and applying multivariate statistics to large (open) datasets, an approach which we follow here as well. FLEXDIST constitutes a promising instrument in the toolbox of comparative research into lithic artefacts in conjunction with other methods aimed at studying complex variation such as geometric morphometrics.

## Materials and methods

### Compiling the lithic flake dataset

The site of Sibhudu is a large rockshelter located in KwaZulu-Natal, South Africa, about 40 km north of Durban. Ongoing excavations since the 1990s headed first by L. Wadley and since 2011 by N. Conard have uncovered a more than 4 m sequence of stratified archaeological deposits of the MSA dated by OSL to >100-38 ka^34–40,64^. Due to the long occupation sequence, the richness of the archaeological material, favourable organic preservation and outstanding findings, Sibhudu has become one of the key sites in Africa to study the early cultural evolution of *Homo sapiens* (e.g.,^66–69^). The massive Sibhudan sequence lies in the upper part of the Sibhudu depositional sequence and dates to ca. 58 ka. Previous techno-typological findings on the lithic assemblages recovered from this part as well as contextual information of these archaeological deposits are described in detail in Supplementary Note S1, Supplementary Table S1 and Supplementary Table S2.

For this study, we selected 16 attributes (see Table 1) recorded for flakes from the Sibhudan sequence from a larger set of variables (*n*=25). These attributes were chosen based on the following criteria: i.) they are commonly employed for assessing variation in lithic artefacts; ii.) they feature various dimensions of flakes and different measurement scales; and iii.) they may be replicable between researchers (e.g.,^30^). We then searched the lithic database from Sibhudu for flakes using the following filtering and data quality criteria: i.) the blank type was designated as flake, excluding blades or bladelets; ii.) clear assignment to one of the four cultural phases in the Sibhudan sequence; iii.) clear characterization by 16 pre-defined mixed continuous, ordinal and nominal variables; and iv.) no more than 20% missing values (a threshold set arbitrarily), corresponding to a maximum of 3 out of 16 variables missing. Flakes with unclear phase attribution, rare morphologies, or more than 20% missing values were excluded from the analysis. The final dataset filtered in this way comprised *n*=4,512 flakes, with *n*=761 belonging to phase 1, *n*=1,689 to phase 2, *n*=594 to phase 3, and *n*=1,468 to phase 4. All raw data used for the flakes and attributes are available in Supplementary Data S1.

Five continuous measurements characterising the overall geometric properties of the flakes (length, width, thickness, platform width, and platform thickness) were converted into scale-free shape variables by dividing each measurement by the geometric mean for all five measurements in each flake. This procedure removes gross size from the data to assess differences in the proportionate contribution of individual variables to overall artefact size, thereby making flakes of different sizes but similar shapes more comparable.

## Quantifying resemblance among flakes

As an initial step in quantifying the resemblance among flakes, we examined the correlation structure of the mixed continuous, ordinal and nominal variables. To achieve this, we computed a heterogeneous correlation matrix among variables using the *mixedCor* function from the *psych* package in R^70^. The heterogeneous correlation matrix computed by *mixedCor* consists of Pearson correlations for continuous variables, polychoric correlations for ordinal variables, tetrachoric correlations for binary variables, and polyserial or biserial correlations for the various mixed variables.

Multivariate morphological resemblance among flakes was quantified using the recently developed FLEXDIST function in R^33^ (available at: https://doi.org/10.5281/zenodo.10591665). Conceptually, the procedure involves four main steps: First, missing values in objects with only partially recorded data are replaced with randomly generated values using a multiple imputation approach, resulting in a large set of differentially imputed datasets. Second, each imputed dataset is submitted to a mixed data PCA to estimate uncorrelated PCs, which are then used to compute an inter-individual Mahalanobis-type distance matrix. Third, these various distance matrices are combined to construct a median pairwise distance matrix (FLEXDIST_median_), along with 95% confidence intervals reflecting the uncertainty resulting from missing values (FLEXDIST_upper_ and FLEXDIST_lower_). Fourth and finally, the first two PCs that explain most of the variation across objects are visualized in a scatterplot (FLEXDIST_plot_). In this plot, each object is represented by a centroid reflecting the average position of its multiple imputed duplicates, with a 95% confidence ellipse around the centroid to show uncertainty arising from missing values. Further methodological details can be found in Rathmann et al.^33^. The FLEXDIST analysis was conducted with 1,000 iterations.

To visually examine the relationships among the original variables and their contributions to the first two PCs displayed in FLEXDIST_plot_, we generated a PCA correlation circle plot. This plot was created using the loadings estimated for the object centroids, reflecting the average positions of their multiple imputed variants. Arrows in the PCA correlation circle plot indicate the direction and strength of each original variable’s contribution to the PCs, with longer arrows representing stronger correlations and directionality showing associations with specific principal axes.

To visually assess differences in intra-phase dispersion, we utilised the information provided in FLEXDIST_median_ and estimated the distance of each flake to the respective group centroid of each phase. We then visualized the resulting distributions of these distance values using the *geom_sina* function from the *ggforce* package in R^71^. In these plots, lower overall values indicate greater homogeneity, while higher overall values reflect increased heterogeneity. For all graphics we used the R functions described above and the *ggplot2* package^72^.

### Statistical inference with PERMANOVA and PERMDISP

We employed two complementary statistical approaches to formally test whether the pattern of morphological resemblance among flakes, as inferred from FLEXDIST_plot_, varied significantly across the four phases at Sibhudu. Unlike the visual inspection of FLEXDIST_plot_, which depicts variation only along the first two PCs, these statistical tests utilise the entire high-dimensional space. First, we conducted permutational multivariate analysis of variance (PERMANOVA)^41^ on the FLEXDIST_median_ distance matrix to test the null hypothesis of no differences in group centroids among phases. A rejection of the null hypothesis in PERMANOVA indicates that the centroids differ between the phases. Like ordinary MANOVA, PERMANOVA partitions the sum of squares between and within groups and uses a pseudo-*F* test to compare between-group to within-group variance. We contrasted the four phases in a pairwise comparison, applying a Bonferroni correction for multiple testing. While PERMANOVA has demonstrated robustness to heterogeneity among groups for balanced designs, it may not hold for unbalanced designs^73^. Thus, in each pairwise comparison, we ensured equal sample sizes by randomly down-sampling the larger sample to match the size of the smaller one. PERMANOVA analysis was conducted using the *adonis2* function implemented in the R package *vegan*^74^, with statistical significance determined after 1,000 permutations. Second, using the down-sampled data, we employed a permutational multivariate analysis of dispersion (PERMDISP)^42^ on the same FLEXDIST_median_ distance matrix to test the null hypothesis of no differences in within-group dispersion among phases. A rejection of the null hypothesis in PERMDISP indicates that dispersion differs between the phases. PERMDISP is a multivariate analog of Levene’s test and compares within-group dispersion based on the average distance of each object to the respective group centroid. Similar to PERMANOVA, we compared the four phases in a pairwise manner, applying a Bonferroni correction for multiple testing. PERMDISP analysis was performed using the *betadisper* and *permutest* functions within the R package *vegan*^74^, with statistical significance determined after 1,000 permutations. To ensure the reproducibility of the down-sampling, we set a random seed using the *set.seed* function, with a seed value of 1.

## Supplementary Information

Supplementary Information 1.

Supplementary Information 2.

Supplementary Information 3.

## Data Availability

All data generated or analysed during this study are included in this published article, its supplements or online repositories. Data: Supplementary Information Data S1 Code: Supplementary Information Code S1.
