# Supplementary material for: Exploring the utility of unretouched lithic flakes as markers of cultural change
Source: Sci Rep. 2025 Jan 10;15:1571. doi: 10.1038/s41598-025-85399-z (PMC11723925; doi:10.1038/s41598-025-85399-z)
Supplement: Supplementary file 3 — Supplementary Information 3. [file 41598_2025_85399_MOESM3_ESM.pdf]

Supplementary Information

Exploring the utility of unretouched lithic flakes as markers of cultural change

Manuel Will, Hannes Rathmann

Content

Note S1. Diachronic cultural change in the Sibhudan sequence and phase distinction 2

Table S1. Overview of the Sibhudan sequence from Sibhudu deriving from the University of Tübingen excavations since 2021. 4

Table S2. Summary of the most important diachronic changes in lithic technology within the Sibhudan sequence (layers RB-BSP) and its four phases 5

Figure S1. Morphological resemblance among 4,512 unretouched lithic flakes from four successive cultural phases at Sibhudu, with each flake characterized by 16 variables (without size-correction applied to overall flake geometry measurements) and allowing for up to 20% missing data. 9

Table S3. Pairwise PERMANOVA and PERMDISP tests conducted on 4,512 unretouched lithic flakes from four cultural phases at Sibhudu, with each flake characterized by 16 variables (without size-correction applied to overall flake geometry measurements) and allowing for up to 20% missing data 10

Figure S2. Morphological resemblance among 4,512 unretouched lithic flakes from five raw material types at Sibhudu, with each flake characterized by 15 variables (excluding the raw material variable) and allowing for up to 20% missing data 11

Table S4. Pairwise PERMANOVA and PERMDISP tests conducted on 4,512 unretouched lithic flakes from five raw material types at Sibhudu, with each flake characterized by 15 variables (excluding the raw material variable) and allowing for up to 20% missing data 12

Figure S3. Morphological resemblance among 4,512 unretouched lithic flakes from four successive cultural phases at Sibhudu, with each flake characterized by 15 variables (excluding the raw material variable) and allowing for up to 20% missing data 13

Table S5. Pairwise PERMANOVA and PERMDISP tests conducted on 4,512 unretouched lithic flakes from four cultural phases at Sibhudu, with each flake characterized by 15 variables (excluding the raw material variable) and allowing for up to 20% missing data 14

Figure S4. Morphological resemblance among 3,189 unretouched lithic flakes from four successive cultural phases at Sibhudu, with each flake characterized by 15 variables (excluding the raw material variable), focusing solely on dolerite, and allowing for up to 20% missing data 15

Table S6. Pairwise PERMANOVA and PERMDISP tests conducted on 4,512 unretouched lithic flakes from four cultural phases at Sibhudu, with each flake characterized by 15 variables (excluding the raw material variable), focusing solely on dolerite, and allowing for up to 20% missing data 16

Supplementary References.. 17

Note S1. Diachronic cultural change in the Sibhudan sequence and phase distinction.

Sibhudu constitutes one of the key sequences of the MSA in South Africa with stratified deposits of occupations rich in archaeological material left by modern humans in a long sequence dating to >100-38 ka [1-3]. The site features a succession of different technocomplexes in its close to 5-m thick stratigraphic sequence, from bottom to top including a basal unnamed MSA, followed by the Ilembian, 'pre-SB', SB, HP, "post-HP"/Sibhudan, late MSA, and final MSA strata [1-6]. The "post-HP" was later renamed to the Sibhudan [7] and is one of the thickest and richest deposits for this period in southern Africa. The depositional sequence of 1.2 m thickness includes a succession of 23 finely laminated find horizons or archaeological layers which are often centimeter thin and almost entirely of anthropogenic origin [see Table S1; 8-10]. This sequence has an extraordinarily high temporal resolution for the Stone Age, with occupations at the base, middle, and top of the deposits dated indistinguishably to ~58 ka by Optically Stimulated Luminescence (see Table S1; weighted mean age of ~58.4 ± 1.4 ka; [4]). Adding the fine lamination of the deposits and the high density of archeological materials to the chronometric data, these observations attest to multiple intense and repeated occupations by MSA hunter-gatherers over the course of only a couple of centuries or few millennia at most [1,2,4,6].

In total, the Sibhudan sequence encompasses 5 m³ sediments, ~200,000 stone tools with very high densities (5,000–90,000 n/m³ lithics), frequent ochre, charcoal and bone, and some bone tools. The richness of and diversity within the material culture coupled with the high-resolution stratigraphy, numerous absolute chronometric dates, multi-proxy environmental and contextual data, and lithic assemblages studied in a systematic and quantitative manner render Sibhudu an ideal case as a type locality for the Sibhudan technocomplex (see [7,11]). In terms of site use, high densities of lithic remains with complete reduction sequences [7,10], abundant traces of transported and butchered animals of various sizes [12,13], and frequent evidence of stacked hearths and other forms of site use and maintenance creating domestic space within a thick and finely laminated sequence [8,9,14] accumulating over short time spans [1,4] suggest the repeated and intense use of Sibhudu as a residential site, likely as a focal point of human occupations for the entire region.

The Sibhudan deposits feature a high number of lithics >3 cm (n=10,882) and <3 cm (n=161,847). The large number of lithic artefacts, highly resolved chronology and similar residential character of the occupations within this sequence provide an ideal setting for studying short-term diachronic cultural change at a site and within a technocomplex, minimizing other factors that also influence lithic technology (e.g., ecological changes, site function or raw material availability). We previously studied the large lithic assemblages >3 cm (n=10,882) of the so-called 'Sibhudan' sequence in this site with the results published in three sequential articles ([10,11,15]; see Table S1). The original study by a single observer included a quantitative and qualitative assessment of all assemblage elements, including blanks (n=9,616), cores (n=164) and tools (n=835). Regarding the methods of these previous lithic analyses, we studied all stone artifacts >30 mm – as well as all cores and tools regardless of size – individually through a combination of multiple methods, drawing from German, French, North American and South African traditions of lithic analyses. The analyses combined information from reduction sequence analyses of entire assemblages and raw material units [16-18] with data from attribute analysis of individual artifacts [19-23]. We employed typological approaches [24,25] – with particular consideration of South African tool taxonomies [26,27] – but also techno-functional analyses of retouched pieces [28-30] to obtain comparable data on tool manufacture across sites and regions. We previously classified tools based on the identification of specific patterns of repetitive retouch on different parts of the tool which indicate distinct retouching sequences [7,11]. The four main techno-functional tool classes and reduction sequences at Sibudu comprise Tongatis, Ndwedwes, naturally backed tools (NBTs), and asymmetric convergent tools (ACTs). In combination, these methods were used to holistically reconstruct technological strategies of the individual assemblages by providing information on the procurement of raw materials and their reduction sequences, the methods of core reduction, the techniques of blank production, and the approach used for tool manufacture and recycling.

The text following this paragraph and Table S2 provide a detailed summary of the identified changes throughout the sequence. These temporal changes include various lithic domains (sensu [31]), including core reduction, blank production and tool manufacture and are predominantly of gradual nature. As such, the Sibhudan sequence at Sibhudu is characterized by both consistent features and diachronic variability, which led to the sub-division of informal phases (or facies) at the site within the same cultural-taxonomic unit. Based on a reading of the entire stratigraphy [10] the Sibudan technocomplex was divided into four phases ('Upper/Classic', 'Upper middle', 'Lower middle' and 'Lower' Sibudan), reflecting the techno-typological variability encountered within the high-resolution sequence (Table S1). The four phases (here Phase 1-4) each exhibit common techno-typological features and gradually build upon another [10,32].

These changes throughout the sequence are incremental and often cumulative in nature, without showing any significant breaks or an overall discontinuous pattern. In accordance with the OSL dates, the nature of these short-term changes at Sibhudu in multiple independent techno-typological traits likely reflect the intergenerational transmission of cultural information (see discussion in [10,15]).

The following elements unite all [or most] assemblages classified as Sibhudan at Sibhudu. Raw material procurement is predominantly local with the use of various rock types of different granularity and knapping qualities [dolerite, hornfels, quartzite, sandstone, quartz]. Knappers used multiple reduction methods alongside each other within each assemblage, encompassing Levallois, discoid, platform, and bipolar methods. The frequency of each method varies markedly throughout the sequence. In terms of blank production, [convergent] flake and blade assemblages characterize the Sibhudan. There is a marked dichotomy in the knapping technique to manufacture blank types: Flakes and convergent flakes are predominantly produced by internal hard stone hammer percussion, whereas blades are knapped with soft stone hammers (i.e., sandstone). The phases are units that are united by distinct characteristics as follows.

Phase 1

The "Upper" or "Classic" Sibhudan constitutes the uppermost phase of the technocomplex and includes its original definition based on 6 archaeological layers (the Sibhudu assemblage type [7,11]). In addition to elements cited above that unite the assemblages, these uppermost Sibudan layers are characterized by a consistently high proportion of unifacial points (38–54%) and retouched pieces in general (17–27%), frequent use of non-local fine-grained hornfels (>20%), relatively high abundance of blades (11–20%) and platform cores for blade production. They also feature the occurrence of four techno-functional tool classes in high proportions (Tongati tool, Ndwedwe tool, Asymmetric convergent tool (ACT), Naturally backed tool) making up over two thirds of all modified pieces. Tongatis and Ndwedwes are particularly frequent in the "Classic" Sibhudan – making up over more than half of all retouched pieces – and constitute a hallmark of these assemblages.

Phase 2

The "Upper middle" Sibhudan directly below the "Classic" Sibhudan encompasses three archaeological layers [15]. The supplemental features of this phase encompass a strong focus on the use of local dolerite, high abundance of discoid reduction, tool assemblages featuring both frequent unifacial points (42%) but also notches and denticulates (~5%), much lower tool frequencies (3–6%) and the highest lithic find densities among the sequence. The assemblages exhibit all techno-functional tool classes, including the first appearance of frequent Tongatis, Ndwedwes and ACTs (total proportion ~30–50%) which increase from bottom to top but remain less abundant compared to the "Classic" Sibhudan.

Phase 3

The ensuing "Lower middle" Sibhudan includes two archaeological layers [15], and features the most frequent use of Levallois reduction in the sequence, frequent notches and denticulates (22–56%), rare unifacial points (n=1), an absence of ACTs, Tongatis and Ndwedwes, and the overall lowest proportions of retouched pieces (~1%). Dolerite continues to be the most frequent raw material.

Phase 4

The "Lower" Sibhudan constitutes the lowermost unit of the Sibhudan directly on top of the Howiesons Poort and was the last to be defined [10]. This phase encompasses a total of 12 layers and shows as additional features high proportions of sandstone and quartz use, frequent bipolar and informal core reduction strategies, predominantly marginal retouch, and the lowest proportion of blades (mean: 4%) and lithic densities among the sequence. The presence of small, invasively-shaped bifacial points (n=14), mostly on quartz, from a total of four layers and the absence of unifacial points and all four techno-functional tool classes in many layers are further characteristics of this phase.

Table S1. Overview of the Sibhudan sequence from Sibhudu deriving from the University of Tübingen excavations since 2021. First mentioned layers designate the bottom of the subdivisions.

Layers	Number of layers	Number of lithic artifacts studied	Informal designation	Primary publications	Chronometric ages[a] (Layer in brackets)
**BM–BSP**	6	2,651	Phase 1: ‘Upper’ or ‘Classic’ Sibudan	[7,11]	57.6 +/- 2.1 (BSP); 59.6 +/- 2.3 (SS);
**SU–POX**	3	4,077	Phase 2: ‘Upper middle’ Sibudan	[15]	59.0 +/- 2.2 (POX)
**SP–WOG1**	2	1,073	Phase 3: ‘Lower middle’ Sibudan	[15]	-
**RB–G1**	12	3,081	Phase 4: ‘Lower’ Sibudan	[10]	58.3 +/- 2.0 (CH2); 58.6 +/- 2.1 (Y1); 58.2 +/- 2.4 (BGMIX)

[a] Absolute ages are from Optically Stimulated Luminescence dating (in ka) and taken from [4]) during Wadley’s excavations.

Table S2. Summary of the most important diachronic changes in lithic technology within the Sibhudan sequence (layers RB-BSP) and its four phases. Domains include raw material procurement, core reduction and preparation, blank production, tool manufacture and lithic density (see e.g., [31]). Color shadings indicate homogeneity in frequency or absence/presence of traits on which phase distinctions were made. Grey color indicates non-relevant traits for phase distinction. Note the frequent overlap of traits along phase transitions.

Phase	Layer	RMU non-local (%)	RMU selection	Core reduction	Blank production	Facet. Platf. (%)	Tool %	Tool classes (n)	Tong. & Ndw. (%)	Unifac. Points / notched (n)	Lithic density (n/m³)
Phase 1	**BSP**	32	1) Dolerite<br>2) Hornfels<br>3) Sandstone	1) Platform laminar<br>2) Parallel/Levallois	1) Flake<br>2) Blade<br>3) Point	27	17	4/4	50	67 / 4	37,400
	**SPCA**	38	1) Dolerite<br>2) Hornfels<br>3) Sandstone	1) Platform laminar<br>2) Parallel/Levallois	1) Flake<br>2) Blade<br>3) Point	23	18	4/4	50	48 / 4	35,300
	**CHE**	38	1) Dolerite<br>2) Hornfels<br>3) Sandstone	1) Platform laminar<br>2) Parallel/Levallois	1) Flake<br>2) Blade<br>3) Point	25	22	4/4	52	11 / 0	32,500
	**MA**	33	1) Dolerite<br>2) Hornfels<br>3) Sandstone	1) Platform laminar<br>2) Parallel/Levallois	1) Flake<br>2) Point<br>3) Blade	22	27	4/4	67	26 / 0	33,800
	**IV**	35	1) Dolerite<br>2) Hornfels<br>3) Sandstone	1) Platform laminar<br>2) Parallel/Levallois	1) Flake<br>2) Blade<br>3) Point	25	27	4/4	58	97 / 11	49,700
	**BM**	25	1) Dolerite<br>2) Hornfels<br>3) Sandstone	1) Platform laminar<br>2) Parallel/Levallois	1) Flake<br>2) Blade<br>3) Point	29	22	4/4	49	29 / 0	40,400
Phase 2	**POX**	6	**1) Dolerite**<br>2) Hornfels<br>3) Sandstone	1) Inclined/Discoid<br>2) Platform laminar	1) Flake<br>2) Blade<br>3) Point	16	6	4/4	28	49 / 17	79,400
	**BP**	3	**1) Dolerite**<br>2) Hornfels<br>3) Sandstone	1) Inclined/Discoid<br>2) Platform laminar	1) Flake<br>2) Point<br>3) Blade	15	3	3/4	44	5 / 0	89,600
	**SU**	1	**1) Dolerite**<br>2) Sandstone<br>3) Quartzite	1) Inclined/Discoid<br>2) Platform laminar	1) Flake<br>2) Blade<br>3) Point	12	3	4/4	10	14 / 10	71,200
Phase 3	**SP**	1	1) Dolerite<br>2) Sandstone<br>3) Quartzite	1) Parallel/Levallois<br>2) Inclined/Discoid	1) Flake<br>2) Point<br>3) Blade	23	1	1/4	0	0 / 7	24,900
	**WOG1**	0	1) Dolerite<br>2) Sandstone<br>3) Quartzite	1) Parallel/Levallois<br>2) Inclined/Discoid	1) Flake<br>2) Point<br>3) Blade	23	1	1/4	0	1 / 2	13,700
Phase 4	**G1**	1	**1) Sandstone**<br>2) Dolerite<br>**3) Quartz**	1) Non-formal<br>2) Parallel/Levallois	1) Flake<br>2) Point<br>3) Blade	23	2	1/4	0	0/0	5,500
	**CH2**	0	**1) Sandstone**<br>2) Dolerite<br>**3) Quartz**	1) Non-formal	1) Flake<br>2) Point<br>3) Blade	16	1	0/4	0	0/0	5,700

	**Y1**	1	1) Dolerite<br>**2) Sandstone**<br>**3) Quartz**	1) Non-formal<br>2) Bipolar	1) Flake<br>2) Point<br>3) Blade	12	2	0/4	0	0/2	10,700
	**BMIX**	1	1) Dolerite<br>**2) Sandstone**<br>**3) Quartz**	1) Non-formal<br>2) Bipolar	1) Flake<br>2) Point<br>3) Blade	16	1	1/4	0	0/3	17,400
	**BBGM**	0	1) Dolerite<br>**2) Sandstone**<br>3) Quartzite	1) Non-formal<br>2) Bipolar	1) Flake<br>2) Point<br>3) Blade	20	2	0/4	0	1/3	14,100
	**YA**	1	1) Dolerite<br>**2) Sandstone**<br>**3) Quartz**	1) Non-formal<br>2) Bipolar	1) Flake<br>2) Point<br>3) Blade	22	3	0/4	0	0/3	19,400
	**YA2**	1	**1) Sandstone**<br>2) Dolerite<br>**3) Quartz**	1) Non-formal<br>2) Bipolar	1) Flake<br>2) Point<br>3) Blade	13	2	1/4	0	0/3	20,100
	**GM**	2	**1) Sandstone**<br>**2) Quartz**<br>3) Dolerite	1) Non-formal<br>2) Bipolar	1) Flake<br>2) Point<br>3) Blade	15	1	0/4	0	0/1	14,800
	**YA2i**	5	**1) Sandstone**<br>**2) Quartz**<br>3) Dolerite	1) Non-formal<br>2) Bipolar	1) Flake<br>2) Point<br>3) Blade	10	4	1/4	0	0/2	12,400
	**BYA2i**	5	**1) Sandstone**<br>**2) Quartz**<br>3) Dolerite	1) Non-formal<br>2) Bipolar	1) Flake<br>2) Blade<br>3) Point	16	2	1/4	0	0/4	9,200
	**LBYA**	2	**1) Sandstone**<br>2) Dolerite<br>3) Quartzite	1) Non formal<br>2) Bipolar	1) Flake<br>2) Blade<br>3) Point	11	3	0/4	0	0/3	14,600
	**RB**	2	1) Dolerite<br>**2) Sandstone**<br>3) Quartzite	1) Non formal<br>2) Bipolar	1) Flake<br>2) Blade<br>3) Point	15	4	1/4	0	0/4	56,500

Table S2. (Continued)

	Layer	RMU non-local (%)	RMU selection	Core reduction	Blank production	Facet. Platf. (%)	Tool %	Tool classes (n)	Tong. & Ndw. (%)	Unifac. Points / notched (n)	Lithic density (n/m³)
**Phase 1**	**BSP** **SPCA** **CHE** **MA** **IV** **BM**	High (25-38%)	1) Dolerite 2) Hornfels *very frequent* 3) Sandstone	Platform laminar dominates, Parallel/Levallois common	1) Flake 2) Blade 3) Point	Very high (22-29%)	High (17-27%)	All present	High (49-67%)	Unifacial points very frequent, Notched tools rare or absent	Intermediate (33,000-50,000)
**Phase 2**	**POX** **BP** **SU**	Very low (0-6%)	1) Dolerite dominance 2) Hornfels 3) Sandstone	Inclined/Discoid dominates, Platform laminar common		Low (12-16%)	Low (1-6%)		Inter-mediate (10-44%)	Unifacial points frequent, notched tools common	Very high (81,000-90,000)
**Phase 3**	**SP** **WOG1**		1) Dolerite 2) Sandstone 3) Quartzite	Parallel/Levallois dominates, Inclined/Discoid common	1) Flake 2) Point	High (23%)		Only NBTs or none	Absent (0%)	More notched tools than unifacial points	Low (14,000-25,000)
**Phase 4**	**G1**		1) Sandstone (dominant		1) Flake						Very low -Low (6,000-20,000)

	**CH2**<br><br>**Y1**<br><br>**BMIX**<br><br>**BBGM**<br><br>**YA**<br><br>**YA2**<br><br>**GM**<br><br>**YA2i**<br><br>**BYA2i**<br><br>**LBYA**<br><br>**RB**		7/12)<br>2) Dolerite (dominant. 5/12)<br>3) Quartz<br>4) Quartzite	Non-formal reduction most frequent;<br>Bipolar reduction frequent; Levallois sometimes present sometimes not;<br>No discoid		Low-intermediate (10-22)				Notched tools often most frequent tool types;<br>No unifacial points at all;<br><br>Bifacials in the very lowermost layers	(**Exception RB: Intermediate: 56,000**)

Figure S1. Morphological resemblance among 4,512 unretouched lithic flakes from four successive cultural phases at Sibhudu, with each flake characterized by 16 variables (without size-correction applied to overall flake geometry measurements) and allowing for up to 20% missing data. a: Pairwise correlations among variables, with color gradients indicating the direction and strength of each correlation. Full variable names and abbreviations are provided in Table 1. b: PCA plot generated by FLEXDIST$_{plot}$, illustrating the multivariate distribution of flakes (depicted as dots) in two-dimensional PC space. Each flake is surrounded by a 95% confidence ellipse, displaying uncertainty resulting from missing data; larger ellipses indicate more missing values, while smaller ellipses indicate fewer missing values. Color-coding denotes the cultural phase attribution of each flake (see panel d). For each phase, a centroid estimate marks the central location (depicted as a square), accompanied by a 95% confidence ellipse illustrating the dispersion around the centroid. The inset box displays a bar plot of eigenvalues, where bars denote the variance explained by each PC, with the variance retained by the first two PCs indicated in black. c: PCA correlation circle plot visualizing how much the original variables are correlated with the first two PCs. Each variable is represented by an arrow originating from the center. Arrows pointing close to the principal axes suggest that the variable is well-represented by that particular PC, while the length of the arrows indicates how much each variable contributes to the PCs. d: Sina plots showing the dispersion of flakes within the four phases, estimated as the distance of each flake to the respective phase centroid. Error bars are superimposed on the distributions to display medians (depicted as dots) and 95% interpercentile ranges (depicted as bars). This figure was generated in R using the code provided in Supplementary Information Code S1.

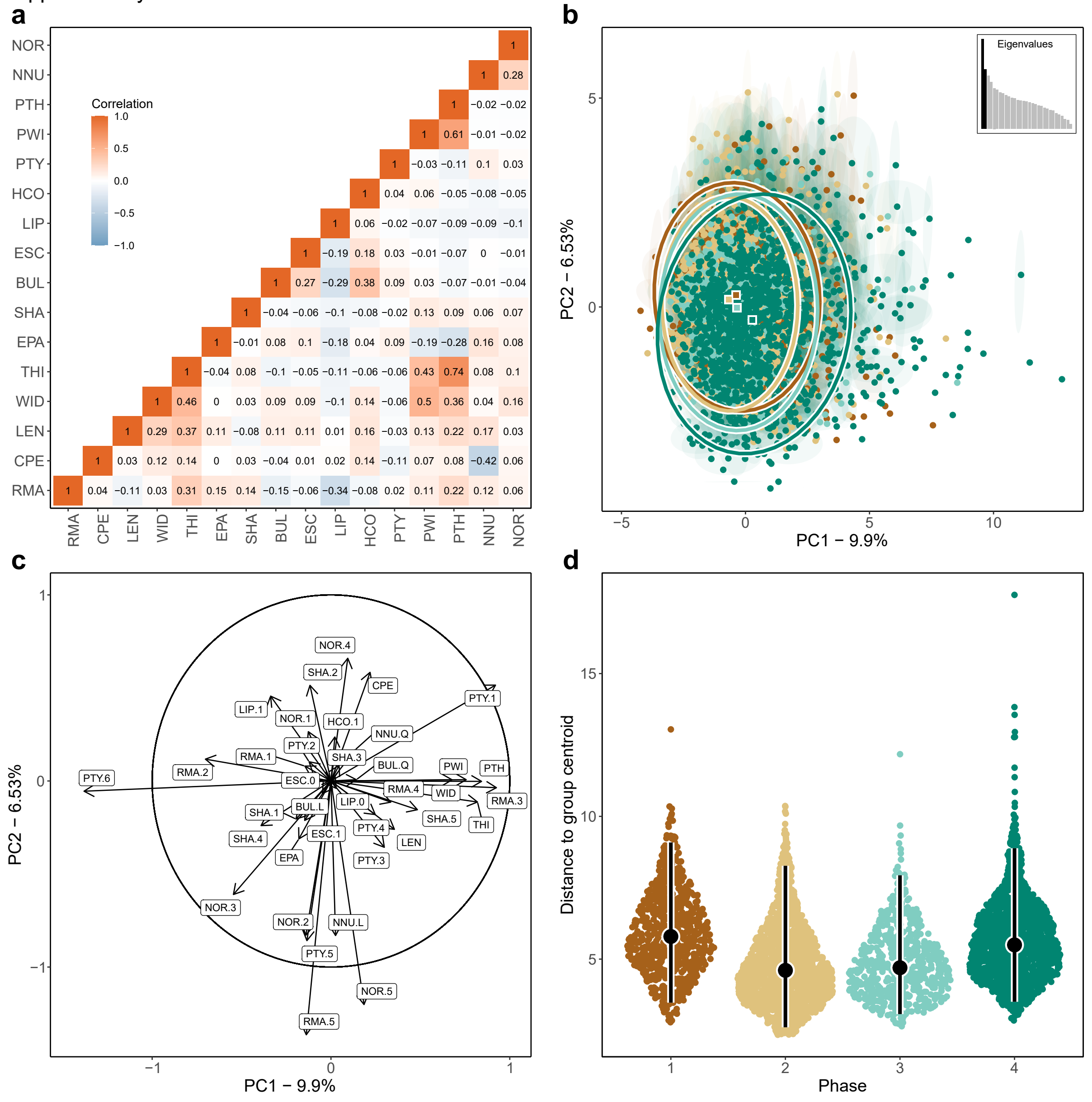

Table S3. Pairwise PERMANOVA and PERMDISP tests conducted on 4,512 unretouched lithic flakes from four cultural phases at Sibhudu, with each flake characterized by 16 variables (without size-correction applied to overall flake geometry measurements) and allowing for up to 20% missing data.

Pairs	PERMANOVA					PERMDISP				
	n	***df***	***F***	***R^2***	***P***	***n***	***df***	***F***	***R^2***	***P***
**Phase 1 vs. 2**	1,522	1; 1,520	18.7070	0.0122	0.0009*	1,522	1; 1,520	190.8747	0.1116	0.0009*
**Phase 1 vs. 3**	1,188	1; 1,186	17.9007	0.0149	0.0009*	1,188	1; 1,186	157.6924	0.1174	0.0009*
**Phase 1 vs. 4**	1,522	1; 1,520	34.0479	0.0219	0.0009*	1,522	1; 1,520	13.4812	0.0088	0.0009*
**Phase 2 vs. 3**	1,188	1; 1,186	4.1830	0.0035	0.0009*	1,188	1; 1,186	0.0004	0.0000	0.9780
**Phase 2 vs. 4**	2,936	1; 2,934	47.8821	0.0161	0.0009*	2,936	1; 2,934	243.2800	0.0766	0.0009*
**Phase 3 vs. 4**	1,188	1; 1,186	15.1546	0.0126	0.0009*	1,188	1; 1,186	88.3781	0.0694	0.0009*

n=sample size; equal sample sizes were ensured by randomly down-sampling the larger sample to match the size of the smaller one; *df*=model and residual degrees of freedom; *F*=*F* test statistic; R^2=coefficient of determination; *P*=*P*-value obtained using 1,000 permutations; *=statistical significance after Bonferroni correction for multiple testing.

Figure S2. Morphological resemblance among 4,512 unretouched lithic flakes from five raw material types at Sibhudu, with each flake characterized by 15 variables (excluding the raw material variable) and allowing for up to 20% missing data. a: Pairwise correlations among variables, with color gradients indicating the direction and strength of each correlation. Full variable names and abbreviations are provided in Table 1. Five continuous measurements characterizing the overall flake geometry (LEN=length, WID=width, THI=thickness, PWI=platform width, and PTH=platform thickness) were converted into scale-free shape variables to enable comparisons among flakes of different sizes but similar shapes. b: PCA plot generated by $FLEXDIST_{plot}$, illustrating the multivariate distribution of flakes (depicted as dots) in two-dimensional PC space. Each flake is surrounded by a 95% confidence ellipse, displaying uncertainty resulting from missing data; larger ellipses indicate more missing values, while smaller ellipses indicate fewer missing values. Color-coding denotes the raw material attribution of each flake (see panel d). For each raw material type, a centroid estimate marks the central location (depicted as a square), accompanied by a 95% confidence ellipse illustrating the dispersion around the centroid. The inset box displays a bar plot of eigenvalues, where bars denote the variance explained by each PC, with the variance retained by the first two PCs indicated in black. c: PCA correlation circle plot visualizing how much the original variables are correlated with the first two PCs. Each variable is represented by an arrow originating from the center. Arrows pointing close to the principal axes suggest that the variable is well-represented by that particular PC, while the length of the arrows indicates how much each variable contributes to the PCs. d: Sina plots showing the dispersion of flakes within the five raw material types, estimated as the distance of each flake to the respective group centroid. Error bars are superimposed on the distributions to display medians (depicted as dots) and 95% interpercentile ranges (depicted as bars). This figure was generated in R using the code provided in Supplementary Information Code S1.

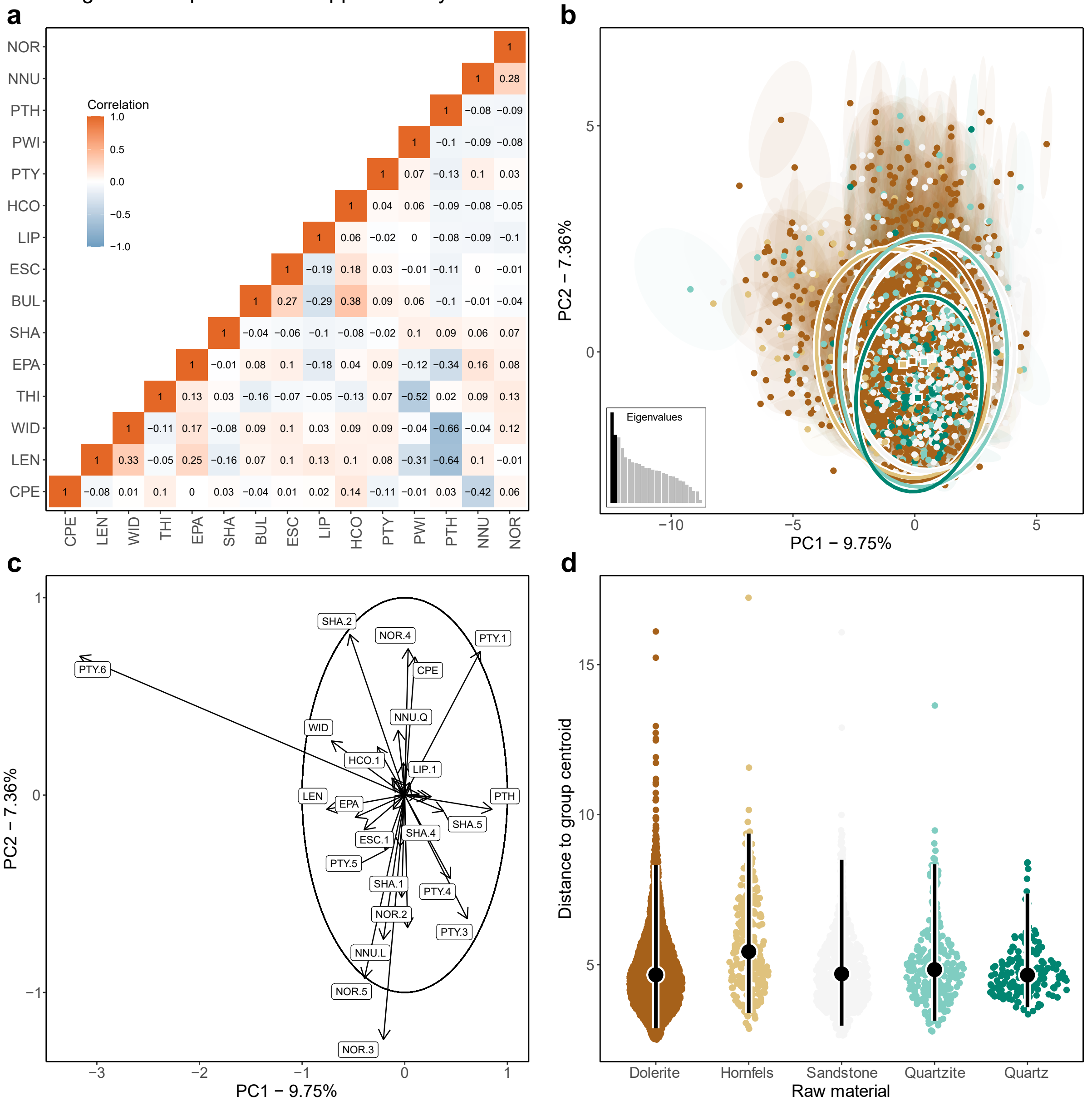

Table S4. Pairwise PERMANOVA and PERMDISP tests conducted on 4,512 unretouched lithic flakes from five raw material types at Sibhudu, with each flake characterized by 15 variables (excluding the raw material variable) and allowing for up to 20% missing data.

Pairs	PERMANOVA					PERMDISP				
	n	***df***	***F***	R^2	***P***	***n***	***df***	***F***	R^2	***P***
**Raw Material 1 vs. 2**	526	1; 524	6.1425	0.0116	0.0009*	526	1; 524	37.4947	0.0668	0.0009*
**Raw Material 1 vs. 3**	1,276	1; 1,274	19.6143	0.0152	0.0009*	1,276	1; 1,274	0.8840	0.0007	0.3836
**Raw Material 1 vs. 4**	500	1; 498	5.2467	0.0104	0.0009*	500	1; 498	1.8263	0.0037	0.1918
**Raw Material 1 vs. 5**	344	1; 342	17.8262	0.0495	0.0009*	344	1; 342	0.2721	0.0008	0.5924
**Raw Material 2 vs. 3**	526	1; 524	16.1471	0.0299	0.0009*	526	1; 524	35.6248	0.0637	0.0009*
**Raw Material 2 vs. 4**	500	1; 498	11.0353	0.0217	0.0009*	500	1; 498	20.0267	0.0387	0.0009*
**Raw Material 2 vs. 5**	344	1; 342	21.5693	0.0593	0.0009*	344	1; 342	38.0377	0.1001	0.0009*
**Raw Material 3 vs. 4**	500	1; 498	3.8086	0.0076	0.0009*	500	1; 498	2.0063	0.0040	0.1628
**Raw Material 3 vs. 5**	344	1; 342	11.4625	0.0324	0.0009*	344	1; 342	0.1600	0.0005	0.6803
**Raw Material 4 vs. 5**	344	1; 342	12.2785	0.0347	0.0009*	344	1; 342	3.8146	0.0110	0.0549

Raw material 1=Dolerite; Raw Material 2=Hornfels; Raw Material 3=Sandstone; Raw Material 4=Quartzite; Raw Material 5=Quartz; *n*=sample size; equal sample sizes were ensured by randomly down-sampling the larger sample to match the size of the smaller one; *df*=model and residual degrees of freedom; *F*=*F* test statistic; R^2=coefficient of determination; *P*=*P*-value obtained using 1,000 permutations; *=statistical significance after Bonferroni correction for multiple testing.

Figure S3. Morphological resemblance among 4,512 unretouched lithic flakes from four successive cultural phases at Sibhudu, with each flake characterized by 15 variables (excluding the raw material variable) and allowing for up to 20% missing data. a: Pairwise correlations among variables, with color gradients indicating the direction and strength of each correlation. Full variable names and abbreviations are provided in Table 1. Five continuous measurements characterizing the overall flake geometry (LEN=length, WID=width, THI=thickness, PWI=platform width, and PTH=platform thickness) were converted into scale-free shape variables to enable comparisons among flakes of different sizes but similar shapes. b: PCA plot generated by $FLEXDIST_{plot}$, illustrating the multivariate distribution of flakes (depicted as dots) in two-dimensional PC space. Each flake is surrounded by a 95% confidence ellipse, displaying uncertainty resulting from missing data; larger ellipses indicate more missing values, while smaller ellipses indicate fewer missing values. Color-coding denotes the cultural phase attribution of each flake (see panel d). For each phase, a centroid estimate marks the central location (depicted as a square), accompanied by a 95% confidence ellipse illustrating the dispersion around the centroid. The inset box displays a bar plot of eigenvalues, where bars denote the variance explained by each PC, with the variance retained by the first two PCs indicated in black. c: PCA correlation circle plot visualizing how much the original variables are correlated with the first two PCs. Each variable is represented by an arrow originating from the center. Arrows pointing close to the principal axes suggest that the variable is well-represented by that particular PC, while the length of the arrows indicates how much each variable contributes to the PCs. d: Sina plots showing the dispersion of flakes within the four phases, estimated as the distance of each flake to the respective phase centroid. Error bars are superimposed on the distributions to display medians (depicted as dots) and 95% interpercentile ranges (depicted as bars). This figure was generated in R using the code provided in Supplementary Information Code S1.

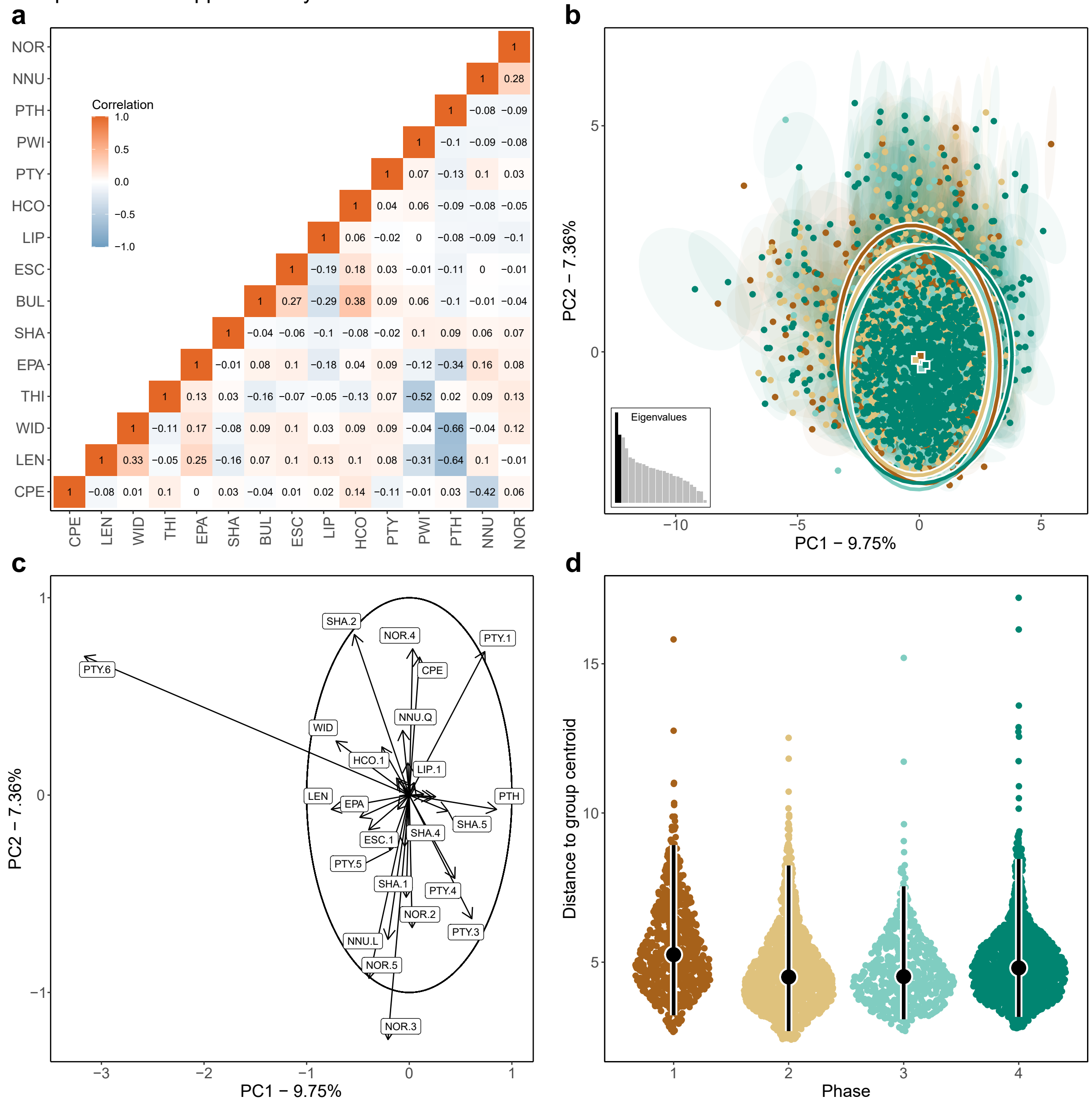

Table S5. Pairwise PERMANOVA and PERMDISP tests conducted on 4,512 unretouched lithic flakes from four cultural phases at Sibhudu, with each flake characterized by 15 variables (excluding the raw material variable) and allowing for up to 20% missing data.

Pairs	PERMANOVA					PERMDISP				
	n	***df***	***F***	R^2	***P***	***n***	***df***	***F***	R^2	***P***
**Phase 1 vs. 2**	1,522	1; 1,520	8.8745	0.0058	0.0009*	1,522	1; 1,520	95.2909	0.0590	0.0009*
**Phase 1 vs. 3**	1,188	1; 1,186	10.9218	0.0091	0.0009*	1,188	1; 1,186	91.5853	0.0717	0.0009*
**Phase 1 vs. 4**	1,522	1; 1,520	23.8051	0.0154	0.0009*	1,522	1; 1,520	58.9874	0.0374	0.0009*
**Phase 2 vs. 3**	1,188	1; 1,186	4.1080	0.0035	0.0009*	1,188	1; 1,186	1.0077	0.0008	0.3117
**Phase 2 vs. 4**	2,936	1; 2,934	34.7848	0.0117	0.0009*	2,936	1; 2,934	23.2364	0.0079	0.0009*
**Phase 3 vs. 4**	1,188	1; 1,186	10.0132	0.0083	0.0009*	1,188	1; 1,186	8.9493	0.0075	0.0020*

n=sample size; equal sample sizes were ensured by randomly down-sampling the larger sample to match the size of the smaller one; *df*=model and residual degrees of freedom; *F*=*F* test statistic; R^2=coefficient of determination; *P*=*P*-value obtained using 1,000 permutations; *=statistical significance after Bonferroni correction for multiple testing.

Figure S4. **Morphological resemblance among 3,189 unretouched lithic flakes from four successive cultural phases at Sibhudu, with each flake characterized by 15 variables (excluding the raw material variable), focusing solely on dolerite, and allowing for up to 20% missing data.** a: Pairwise correlations among variables, with color gradients indicating the direction and strength of each correlation. Full variable names and abbreviations are provided in Table 1. Five continuous measurements characterizing the overall flake geometry (LEN=length, WID=width, THI=thickness, PWI=platform width, and PTH=platform thickness) were converted into scale-free shape variables to enable comparisons among flakes of different sizes but similar shapes. b: PCA plot generated by $\text{FLEXDIST}_{\text{plot}}$, illustrating the multivariate distribution of flakes (depicted as dots) in two-dimensional PC space. Each flake is surrounded by a 95% confidence ellipse, displaying uncertainty resulting from missing data; larger ellipses indicate more missing values, while smaller ellipses indicate fewer missing values. Color-coding denotes the cultural phase attribution of each flake (see panel d). For each phase, a centroid estimate marks the central location (depicted as a square), accompanied by a 95% confidence ellipse illustrating the dispersion around the centroid. The inset box displays a bar plot of eigenvalues, where bars denote the variance explained by each PC, with the variance retained by the first two PCs indicated in black. c: PCA correlation circle plot visualizing how much the original variables are correlated with the first two PCs. Each variable is represented by an arrow originating from the center. Arrows pointing close to the principal axes suggest that the variable is well-represented by that particular PC, while the length of the arrows indicates how much each variable contributes to the PCs. d: Sina plots showing the dispersion of flakes within the four phases, estimated as the distance of each flake to the respective phase centroid. Error bars are superimposed on the distributions to display medians (depicted as dots) and 95% interpercentile ranges (depicted as bars). This figure was generated in R using the code provided in Supplementary Information Code S1.

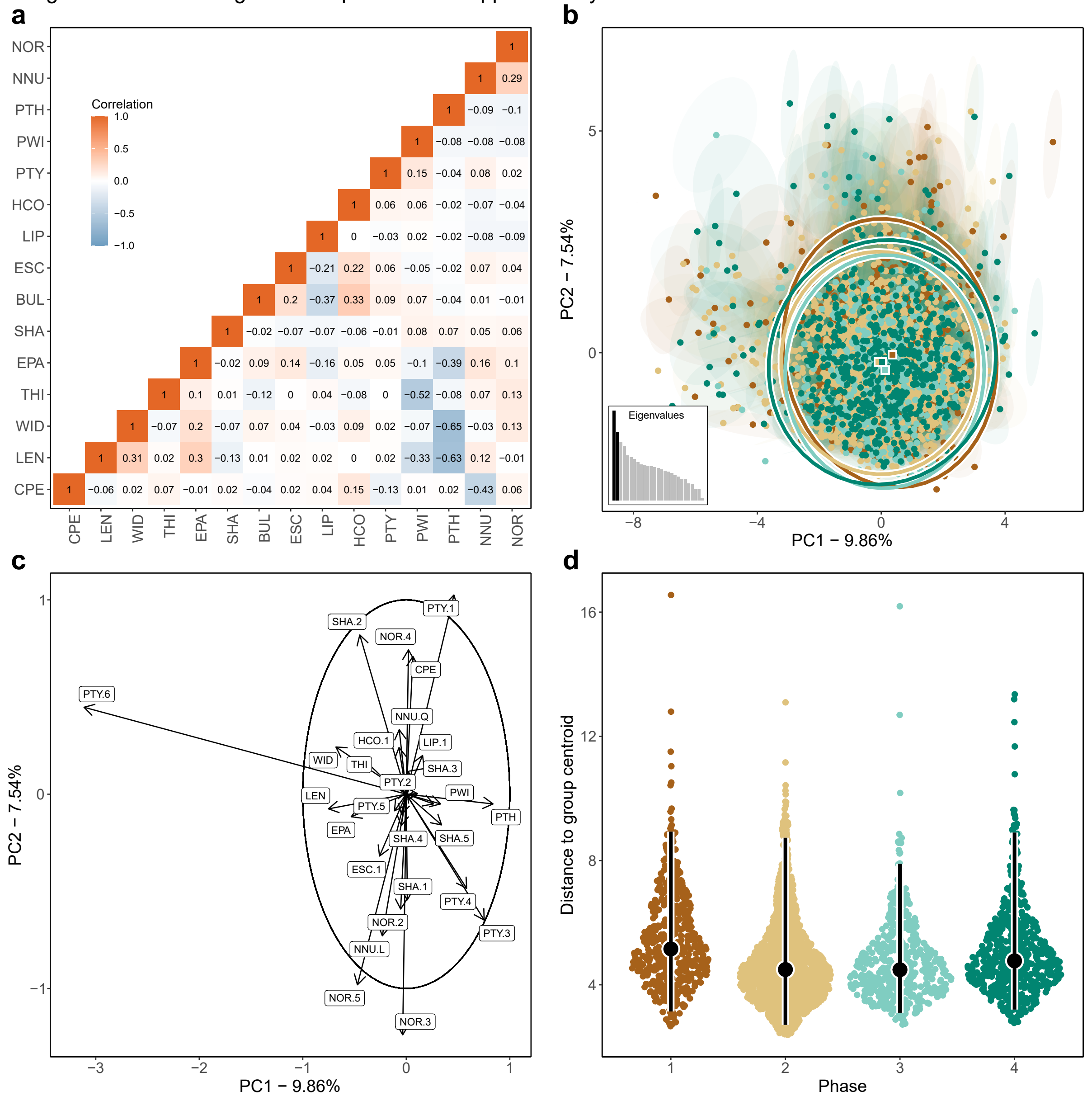

Table S6. **Pairwise PERMANOVA and PERMDISP tests conducted on 4,512 unretouched lithic flakes from four cultural phases at Sibhudu, with each flake characterized by 15 variables (excluding the raw material variable), focusing solely on dolerite, and allowing for up to 20% missing data**.

Pairs	PERMANOVA					PERMDISP				
	n	***df***	***F***	R^2	***P***	***n***	***df***	***F***	R^2	***P***
**Phase 1 vs. 2**	1,060	1; 1,058	5.8353	0.0055	0.0009*	1,060	1; 1,058	52.0299	0.0469	0.0009*
**Phase 1 vs. 3**	1,006	1; 1,004	9.1199	0.0090	0.0009*	1,006	1; 1,004	58.3531	0.0549	0.0009*
**Phase 1 vs. 4**	1,060	1; 1,058	13.9226	0.0130	0.0009*	1,060	1; 1,058	22.2502	0.0206	0.0009*
**Phase 2 vs. 3**	1,006	1; 1,004	4.0481	0.0040	0.0009*	1,006	1; 1,004	0.2072	0.0002	0.6304
**Phase 2 vs. 4**	1,208	1; 1,206	11.3789	0.0093	0.0009*	1,208	1; 1,206	9.8833	0.0081	0.0040*
**Phase 3 vs. 4**	1,006	1; 1,004	7.2574	0.0072	0.0009*	1,006	1; 1,004	12.1808	0.0120	0.0009*

n=sample size; equal sample sizes were ensured by randomly down-sampling the larger sample to match the size of the smaller one; *df*=model and residual degrees of freedom; *F*=*F* test statistic; R^2=coefficient of determination; *P*=*P*-value obtained using 1,000 permutations; *=statistical significance after Bonferroni correction for multiple testing.

Supplementary References

1) Wadley, L., Jacobs, Z., 2006. Sibudu Cave: background to the excavations, stratigraphy and dating. S. Afr. Humanit. 18, 1–26.
2) Wadley, L., 2013. MIS 4 and MIS 3 occupations in Sibudu, KwaZulu-Natal, South Africa. S. Afr. Archaeol. Bull. 68, 41–51.
3) Rots, V., Lentfer, C., Schmid, V. C., Porraz, G., & Conard, N. J. 2017. Pressure flaking to serrate bifacial points for the hunt during the MIS5 at Sibudu Cave (South Africa). PloS one, 12(4), e0175151.
4) Jacobs, Z., Roberts, R.G., Galbraith, R.F., Deacon, H.J., Grün, R., Mackay, A., Mitchell, P.J., Vogelsang R., Wadley, L., 2008. Ages for the Middle Stone Age of southern Africa: implications for human behaviour and dispersal. Science 322, 733–735.
5) Schmid, V. C., Porraz, G., Zeidi, M., & Conard, N. J. 2019. Blade technology characterizing the MIS 5 DA layers of Sibudu Cave, South Africa. Lithic Technology, 44(4), 199-236.
6) Will, M., & Conard, N. J. 2020. Regional patterns of diachronic technological change in the Howiesons Poort of southern Africa. PloS One, 15(9), e0239195.
7) Conard, N. J., Porraz, G., & Wadley, L. (2012). What is in a name?: Characterising the'Post-Howieson's Poort'at Sibudu. South African Archaeological Bulletin, 67(196), 180-199.
8) Goldberg, P., Miller, C. E., Schiegl, S., Ligouis, B., Berna, F., Conard, N. J., & Wadley, L. (2009). Bedding, hearths, and site maintenance in the Middle Stone age of Sibudu cave, KwaZulu-Natal, South Africa. Archaeological and Anthropological Sciences, 1, 95-122.
9) Miller, C.E.M., 2015. High-resolution gearchaeology and settlement dynamics at the Middle Stone Age sites of Diepkloof and Sibudu, South Africa. In: Conard, N.J., Delagnes, A. (Eds.), Settlement dynamics of the Middle Paleolithic and Middle Stone Age, Vol. IV. Kerns Verlag, Tübingen, pp. 27–46
10) Will, M., & Conard, N. J. 2018. Assemblage variability and bifacial points in the lowermost Sibudan layers at Sibudu, South Africa. Archaeological and Anthropological Sciences, 10, 389-414.
11) Will, M., Bader, G.D., Conard, N.J., 2014. Characterizing the Late Pleistocene MSA lithic technology of Sibudu, KwaZulu-Natal, South Africa. PLoS ONE 9, e98359.
12) Clark, J. L. (2013). Exploring the relationship between climate change and the decline of the Howieson’s Poort at Sibudu Cave (South Africa). Zooarchaeology and modern human origins: human hunting behavior during the Later Pleistocene, 9-18.
13) Clark, J.L., Plug. I., 2008. Animal exploitation strategies during the South African Middle Stone Age: Howiesons Poort and post-Howiesons Poort fauna from Sibudu Cave. J. Hum. Evol. 54, 886–898.
14) Wadley, L., Sievers, C., Bamford, M., Goldberg, P., Berna, F., Miller, C., 2011. Middle Stone Age bedding construction and settlement patterns at Sibudu, South Africa. Science 334, 1388–1391
15) Conard, N.J., Will, M., 2015. Examining the causes and consequences of short-term behavioral change during the Middle Stone Age at Sibudu, South Africa. PLoS ONE 10(6), e0130001.
16) Boëda, E., Geneste, J.M., Meignen, L., 1990. Identification de chaînes opératoires lithiques du Paléolithique ancien et moyen. Paléo 2, 43–80.Bonilauri 2010
17) Inizan, M.L., Reduron M., Roche, H., Tixier, J., 1995. Technologie de la pierre taillée. Préhistoire de la pierre taillée, Tome 4. Cercle de recherches et d'études préhistoriques, Meudon.
18) Soressi, M., Geneste, J-M., 2011. The history and efficacy of the chaîne opératoire approach to lithic analysis: studying techniques to reveal past societies in an evolutionary perspective. PaleoAnthropology 2011, 334–350.
19) Hahn, J., 1991. Erkennen und Bestimmen von Stein- und Knochenartefakten: Einführung in die Artefaktmorphologie. Archaeologica Venatoria, Tübingen.
20) Shott, M.J., 1994. Size and form in the analysis of flake debris: review and recent approaches. J. Archaeol. Method Th. 1, 69–110.
21) Tostevin, G.B., 2003. Attribute analysis of the lithic technologies of Stránská skála II–III in their regional and inter-regional context. In: Svoboda, J., Bar-Yosef, O. (Eds.), Stránská skála: origins of the Upper Palaeolithic in the Brno Basin. Peabody Museum Publications, Cambridge (MA), pp. 77–118.
22) Odell, G.H., 2004. Lithic analysis. Manuals in archaeological method, theory, and technique. Kluwer Academic, New York.
23) Andrefsky,W., 2005. Lithics: macroscopic approaches to analysis, 2nd edition. Cambridge University Press, Cambridge
24) Bordes, F,. 1961. Typologie du Paléolithique ancien et moyen. Mémoire n°1. Publications de l'Institut de Préhistoire de l'Université de Bordeaux, Bordeaux.
25) Debénath, A., Dibble, H.L., 1994. Handbook of Paleolithic typology. Vol. I: Lower and Middle Paleolithic of Europe. The University Museum, Philadelphia.
26) Volman, T.P., 1981. The Middle Stone Age in the southern Cape. Unpublished PhD thesis. University of Chicago, Chicago.
27) Wurz, S., 2000. The Middle Stone Age at Klasies River, South Africa. Ph.D. thesis, Stellenbosch University.
28) Boëda, E., 2001. Détermination des unités techno-fonctionnelles de pieces bifaciales provenant de la couche acheuléenne C3 base du site de Barbas I. In: Clique, D. (Ed.), Les industries à outils bifaciaux du Paléolithique Moyen d’Europe occidentale. Liège, E.R.A.U.L., pp. 51–76.
29) Soriano, S., 2001. Statut fonctionnel de l’outillage bifacial dans les industries du Paléolithique moyen: propositions méthodologiques. In: Clique, D. (Ed.), Les industries à outils bifaciaux du Paléolithique Moyen d’Europe occidentale. E.R.A.U.L., Liège, pp. 77–84

30) Bonilauri, S. (2010). Les outils du Paléolithique moyen, une mémoire technique oubliée? Approche techno-fonctionnelle appliquée à un assemblage lithique de conception Levallois provenant du site d'Umm el Tlel (Syrie centrale) (Doctoral dissertation, Paris 10).
31) Tostevin, G.B., 2012. Seeing lithics: a middle-range theory for testing cultural transmission in the Pleistocene. Oxbow Books, Oxford.
32) Will, M. 2019. Sibudan. In Oxford Research Encyclopedia of Anthropology. https://doi.org/10.1093/acrefore/9780190854584.013.35

.
